# Structural and functional aspects of mannuronic acid–specific PL6 alginate lyase from the human gut microbe *Bacteroides cellulosilyticus*

**DOI:** 10.1074/jbc.RA119.010206

**Published:** 2019-09-17

**Authors:** Emil G. P. Stender, Christian Dybdahl Andersen, Folmer Fredslund, Jesper Holck, Amalie Solberg, David Teze, Günther H. J. Peters, Bjørn E. Christensen, Finn L. Aachmann, Ditte H. Welner, Birte Svensson

**Affiliations:** ‡ Department of Biotechnology and Biomedicine, Technical University of Denmark, DK-2800 Kgs. Lyngby, Denmark; § Novo Nordisk Foundation Center for Biosustainability, Technical University of Denmark, DK-2800 Kgs. Lyngby, Denmark; ¶ Department of Biotechnology and Food Science, NTNU, Norwegian University of Science and Technology, N-7491 Trondheim, Norway; ‖ Department of Chemistry, Technical University of Denmark, DK-2800 Kgs. Lyngby, Denmark

**Keywords:** alginate lyase, crystal structure, molecular docking, enzyme kinetics, enzyme mechanism, enzyme mutation, asparagine ladder, Bacteroides cellulolyticus, imidazole rescue, parallel β-helix

## Abstract

Alginate is a linear polysaccharide from brown algae consisting of 1,4-linked β-d-mannuronic acid (M) and α-l-guluronic acid (G) arranged in M, G, and mixed MG blocks. Alginate was assumed to be indigestible in humans, but bacteria isolated from fecal samples can utilize alginate. Moreover, genomes of some human gut microbiome–associated bacteria encode putative alginate-degrading enzymes. Here, we genome-mined a polysaccharide lyase family 6 alginate lyase from the gut bacterium *Bacteroides cellulosilyticus* (*Bcel*PL6). The structure of recombinant *Bcel*PL6 was solved by X-ray crystallography to 1.3 Å resolution, revealing a single-domain, monomeric parallel β-helix containing a 10-step asparagine ladder characteristic of alginate-converting parallel β-helix enzymes. Substitutions of the conserved catalytic site residues Lys-249, Arg-270, and His-271 resulted in activity loss. However, imidazole restored the activity of *Bcel*PL6-H271N to 2.5% that of the native enzyme. Molecular docking oriented tetra-mannuronic acid for *syn* attack correlated with M specificity. Using biochemical analyses, we found that *Bcel*PL6 initially releases unsaturated oligosaccharides of a degree of polymerization of 2–7 from alginate and polyM, which were further degraded to di- and trisaccharides. Unlike other PL6 members, *Bcel*PL6 had low activity on polyMG and none on polyG. Surprisingly, polyG increased *Bcel*PL6 activity on alginate 7-fold. LC–electrospray ionization–MS quantification of products and lack of activity on NaBH_4_-reduced octa-mannuronic acid indicated that *Bcel*PL6 is an endolyase that further degrades the oligosaccharide products with an intact reducing end. We anticipate that our results advance predictions of the specificity and mode of action of PL6 enzymes.

## Introduction

**Figure 1.**
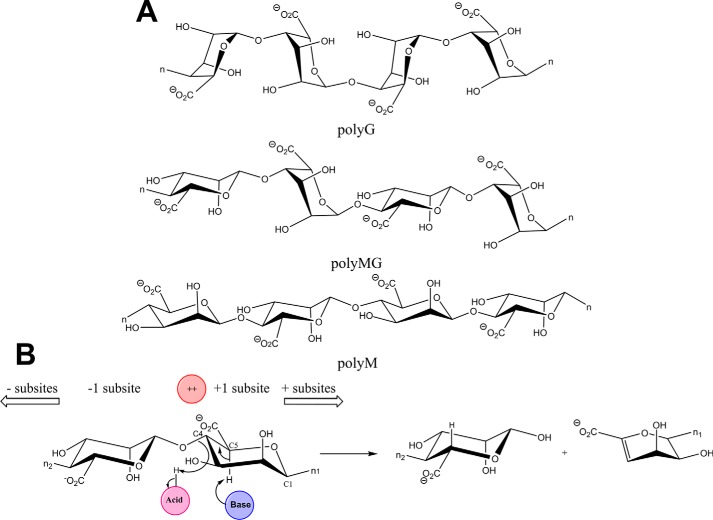
**Schematics illustrating alginate block structures and general lyase mechanism of PL6 enzymes.** *A,* 1,4-linked α-l-guluronic acid block (*top*), 1,4-β-d-mannuronic acid and α-l-guluronic acid mixed linkage block (*middle*), and 1,4-linked β-d-mannuronic acid block (*bottom*). *n* represents the continued polymer. *B, syn*-mechanism of alginate lyases with positions of catalytic Brøndsted acid (*pink*) and base (*blue*) and the uronic acid group neutralizer, ++ (typically Ca^2+^; in *red*).

Alginates are linear anionic polysaccharides present in the cell walls of brown seaweeds. They are composed of blocks of 1,4-linked β-d-mannuronic acid (M),^2^ its C-5 epimer α-l-guluronic acid (G), and of both M and G arranged in alternating or random order (Fig. 1*A*) (1, 2). Alginates are hydrocolloids and serve as gelling and stabilizing agents in food and pharmaceutical products (sodium alginate ref. no. 00148) (4). Moreover, alginates and alginate oligosaccharides have applications in the biomedicine and health sectors (5–7). Biofilms produced by some terrestrial bacteria, *e.g. Azotobacter vinelandii* and *Pseudomonas aeruginosa,* contain alginates *O*-acetylated on C2 and C3 in the M blocks with low G content (8).

Humans lack alginate-degrading enzymes, but certain gut bacteria, *e.g.* strains of the commensal *Bacteroides ovatus*, *Bacteroides xylanisolvens,* and *Bacteroides thetaiotaomicron*, can grow on and ferment alginate *in vitro* to form healthy and beneficial short-chain fatty acids (9–13). The population of Bacteroidetes, Bifidobacteria, and Lactobacilli increased in the gut of rats fed alginate (10), and alginate oligosaccharides were bifidogenic in skim milk media (9). Little is known, however, at the molecular level on alginate breakdown and utilization in the gut beyond the demonstrated substrate specificity of a PL17 enzyme from *Bacteriodes eggerthii*, found to be polyM-specific (14). By contrast, several polysaccharide lyases (PLs) involved in alginate utilization have been described from marine bacteria, including bacteria of the Bacteroidetes phylum (15–17).

PLs are categorized in 37 families in the CAZy database (www.cazy.org)^3^ (3), 10 of which (PL5–7, -14, -15, -17, -18, -32, -34, and -36) contain alginate lyases (18, 19). Alginate lyases break the O–C4 bond to uronic acid residues through a β-elimination reaction that leads to formation of the 4,5-unsaturated sugar 4-deoxy-l-*erythro*-hex-4-enopyranosyluronic acid (denoted as Δ) at the nonreducing end of the released product. Alginate lyases are either endo-acting (2), initially releasing oligosaccharides that can undergo further degradation, typically to di- and trisaccharides (14, 20), or exo-acting producing the unsaturated monosaccharide Δ (Fig. 1*B*) (21, 22). A PL6 family enzyme has yet to be characterized from the gut niche. PL6 is multispecific and can be divided into three subfamilies (19), PL6_1 of endo- and exo-acting alginate or dermatan sulfate–specific enzymes, and PL6_2 and PL6_3, which are reported to contain only polyMG endolyases (20). Most characterized alginate lyases of PL6_1 have broad substrate specificity on polyMG and polyG (20, 23, 24), but a few, *e.g.* Patl3640 and Pedsa0631 from *Pseudoalteromonas atlantica* and *Pseudobacter saltans* respectively, are strictly polyG-specific (20).

With regard to three-dimensional structures, alginate lyases adopt several different folds: β-jelly roll; (α/α)*_n_* toroid; and parallel β-helix, and some are multimodular (21, 22, 25). PL6 displays a right-handed parallel β-helix fold similar to several other polysaccharide lyase families (21, 22, 25). The first PL6 crystal structure was determined for the single domain chondroitin B lyase from *Pedobacter heparinus* DSM 2366 (PBD code 1OFL) that degrades dermatan sulfate (26). Recently, structures also became available for two marine bacterial alginate lyases, namely the polyG-specific homodimeric, two-domain exolyase AlyGC from *Paraglaciecola chatamensis* S18K6T (PDB code 5GKQ) that produces Δ, and the monomeric, single-domain endolyase AlyF from *Vibrio splendidus* OU2 (PDB code 5Z9T), releasing unsaturated trisaccharides from alginates and polyG (27, 28). PL6 thus encompasses various types of specificity toward alginates as well as for dermatan sulfate, an *O*-sulfated glycosaminoglycan of alternating 1,3-β-d-galactosamine and 1,4 α-l-iduronic acid (20, 21). PL6 is proposed to have conserved lysine and arginine residues acting as catalytic residues. This is opposed to alginate lyases of other PL families in which tyrosine and histidine are identified as catalytic residues (21, 22, 25). In PL6, the negatively charged C6 carboxyl group accommodated at subsite +1 is neutralized by Ca^2+^. This reduces the p*K_a_* of the C5 proton facilitating its abstraction by the general base catalyst (Fig. 1*B*) (26, 27, 29, 30). However, a calcium-independent PL6 alginate lyase was reported recently (28). The proton abstraction can occur either in *syn* configuration, having the C5 proton and the glycosidic oxygen of the bond to be cleaved situated on the same side of the sugar ring in the transition state, as is the case of M-specific lyases, or in *anti* configuration when these groups are placed on opposite sides of the sugar ring, as for breaking G-linkages (21, 22). The majority of characterized PL6 members produce di- and tetrasaccharides as end products (20).

Here, we show that *Bcel*PL6 of PL6 subfamily 1 from the human commensal gut bacterium *Bacteroides cellulosilyticus* CRE21 is a monomeric, single-domain polyM-specific enzyme. The crystal structure is solved to 1.3 Å resolution and contains a long, highly-conserved asparagine ladder. The residues at the active site provide insights into specificity determinants in PL6.

## Results

### BcelPL6 homologues in human gut Bacteroides genomes

Most known genes from marine *Bacteroides* associated with alginate utilization, except from PL7, have orthologues in *B. cellulosilyticus* CRE21 as identified by a BLAST search. Searching against nonredundant protein sequences revealed that *Bcel*PL6 is conserved in *Bacteroides* with homologues of >85% sequence identity in strains of human gut *Bacteroides intestinalis*, *Bacteroide*s sp. 14(A), *Bacteroides oleiciplenus*, *Bacteroides timonensis,* and *Bacteroides stercorirosoris*. Although gene up-regulation has not been analyzed for human gut *Bacteroides* growing on alginate, it has been reported in the cases of several members of PL6, PL7, and PL17 from the marine *Gramella forsetti* that belongs to the Bacteroidetes phylum and for *Alteromonas macleodii* (15, 31). *B. cellulosilyticus* of the HGM encodes polysaccharide utilization loci (PULs) involved in degradation and product uptake of polysaccharides, *e.g.* starch (32). *Bcel*PL6 was not annotated to a PUL (32), but *Bcel*PL6 orthologues are predicted along with an annotated PL17 in PULs of *B. intestinalis* DSM 17393, *B. ovatus* NLAE-zl-H73, and *B. xylanisolvens* NLAE-zl-G339 of the HGM (14, 32). A Pfam domain search suggested *Bcel*PL6 is a chondroitinase B, yet another PL6 specificity. This reflects that target substrate variation probably correlates with subtle changes in the active-site structure in PL6 (26–29). Therefore, sequence-based prediction of PL6 specificities is currently not reliable.

### Specificity and mode of action

**Figure 2.**
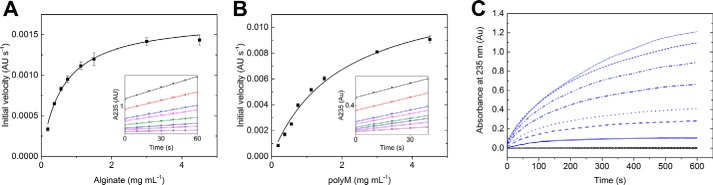
**Kinetics and specificity of *Bcel*PL6.** Michaelis-Menten plots of alginate degraded by 100 nm *Bcel*PL6 (*A*) and polyM degraded by 50 nm *Bcel*PL6 (*B*). *Insets* in *A* and *B* show linear regression of the initial part of the progress curve (from 0.2 mg ml^−1^ (*purple*) to 4.2 mg ml^−1^ (*black*) substrate). *C,* progress of absorbance at 235 nm by action of 300 nm *Bcel*PL6 on 0.2–2.2 mg ml^−1^ polyM (*blue*) or polyG (*black*).

*Bcel*PL6 catalyzed the release of products with unsaturated nonreducing ends (Fig. 1*B*) from alginate (Fig. 2*A*) and polyM (Fig. 2*B*). The reactions followed Michaelis-Menten kinetics, and *k*_cat_ was 8-fold higher for polyM (43.4 ± 1.6 s^−1^) than alginate (*k*_cat_ = 5.4 ± 0.15 s^−1^), whereas *K_m_* was 3-fold lower for alginate (0.59 ± 0.04 mg ml^−1^) than polyM (*K_m_* = 1.96 ± 0.18 mg ml^−1^) (Table 1). Activity was barely detected toward polyG (Fig. 2*C*; Table 1; Fig. S2*A*) and polyMG (Table 1; Fig. S2*B*) even at high concentrations (6 μm) of *Bcel*PL6, and the observed very low rates of degradation of 0–2.0 mg ml^−1^ polyG or polyMG did not follow Michaelis-Menten kinetics (Table 1). Trace of product formation from polyG possibly stems from the 3% M being found in the used polyG candidate substrate. Moreover, *Bcel*PL6 did not degrade acetylated polyM that mimics bacterial alginate (Table 1 and Fig. S2*C*) (8).

**Table 1:** **Kinetic parameters of *Bcel*PL6** Fits to Michaelis-Menten equation (shown in Fig. 2, *A* and *B*) have an *R*^2^ of 0.99 or better. When no value is provided, the initial rates as a function of substrate concentration could not be fitted to a Michaelis-Menten, and instead, the activity on 2 mg ml^−1^ substrate is reported. ND means not detected, and NA means not applicable.

Substrate	*k*_cat_	*K_m_*
	*s*^−*1*^	*mg ml*^−*1*^
Alginate	5.4 ± 0.1	0.58 ± 0.04
PolyM	43.4 ± 1.6	1.96 ± 0.2
Acetylated polyM	ND	ND
PolyG	<0.06*^a^*	NA
PolyMG	<0.08*^a^*	NA

*^a^* Value was reported as activity on 2 mg ml^−1^ substrate.

**Figure 3.**
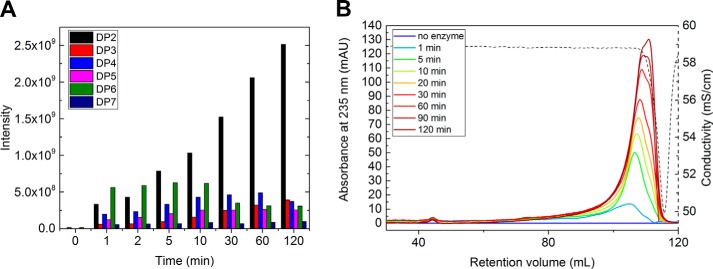
**Progress of products release by *Bcel*PL6 from alginate.** *A,* quantification of reaction products (LC-ESI-MS) during 0–120 min. *B,* size-exclusion chromatography (Superdex 200) of products of degradation from 4 mg ml^−1^ alginate by 100 nm *Bcel*PL6. *Curves* (*blue to brown*) correspond to reaction for 0, 1, 5, 10, 20, 30, 60, 90, and 120 min prior to enzyme inactivation. *Dashed line* is the conductivity, which drops due to low salt in the samples and the SEC-run being complete.

LC-ESI-MS analysis showed that *Bcel*PL6 initially released unsaturated oligosaccharides of DP2–7 from alginate with DP4 and DP6 transiently increasing in abundance and DP2 being the predominant end product (Fig. 3*A*; Table S1). Monosaccharide products were not observed. Molecular masses of released unsaturated oligosaccharides of DP2–7 were confirmed using MALDI-TOF MS (Fig. S3) and quantified by LC-ESI-MS. *Bcel*PL6 released di- and trisaccharides from polyM (Fig. S4) with pentasaccharides dominating initially (Fig. S4; Table S1). Size-exclusion chromatography (SEC) on Superdex 200 that separates linear dextrans of 1–100 kDa showed increasing amounts of unsaturated breakdown products of alginate (*M̄_n_* = 40 kDa) as a broad asymmetric peak containing oligosaccharides (Fig. 3*B*). Thus unsaturated oligosaccharides are the primary products in agreement with the LC-ESI-MS analysis (Fig. 3*A*). As no unsaturated higher molecular weight products were observed (Fig. 3*B*), the mode of action of *Bcel*PL6 can be described as a specific attack on M blocks in alginate followed by further degradation of the released oligosaccharides (20, 23). This is in agreement with both endo- and exo-lyases to occur in PL6_1 (20, 24, 27). Moreover, *Bcel*PL6 seems to recognize the reducing end of the oligosaccharide substrates as it did not further degrade octa-mannuronic acid after NaBH_4_ reduction (Fig. S2*D*).

### PolyG activation of BcelPL6

**Figure 4.**
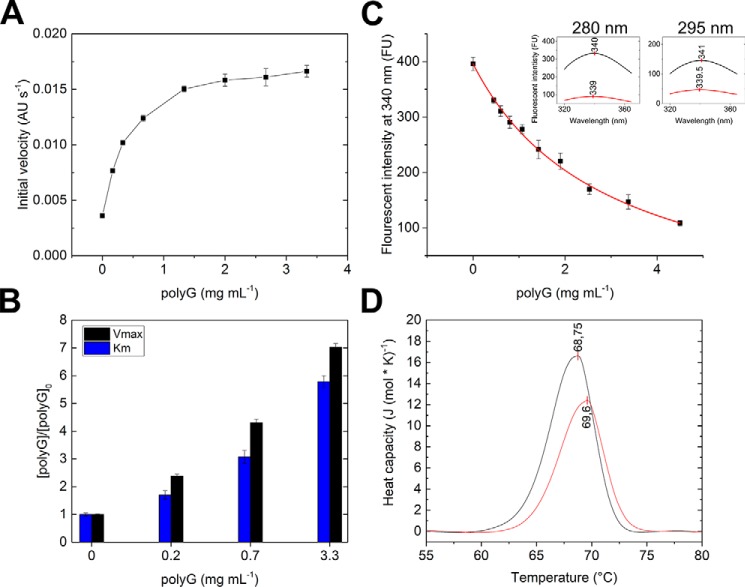
**BcelPL6 activation by polyG.** *A,* initial velocity of 50 nm *Bcel*PL6 degrading 3 mg ml^−1^ alginate in the presence of 0–3.3 mg ml^−1^ polyG. *B,* influence of polyG on *K_m_* (*blue*) and *V*_max_ (*black*) for alginate degradation normalized to values without polyG. *C,* fluorescence intensity of *Bcel*PL6 with 0–4.5 mg ml^−1^ polyG. The *red line* is the fitted binding function. *Insets* are emission scans of *Bcel*PL6 without (*black*) and with (*red*) 4.5 mg ml^−1^ polyG and excitation at 280 nm (*left*) and 295 nm (*right*). The *vertical red line* on scans indicate the fluorescence maximum wavelength. *D,* DSC of *Bcel*PL6 with 5 mg ml^−1^ polyG (*red*) and without (*black*). *Vertical red lines* indicate *T_m_*.

Addition of polyG to *Bcel*PL6 acting on alginate increased the activity up to 5-fold at 3.3 mg ml^−1^ polyG (Fig. 4*A*), with *k*_cat_ and *K_m_* both increasing about 7-fold (Fig. 4*B*). The polyG binding was monitored by decreased fluorescence intensity of *Bcel*PL6 and *F*_max_ blue-shifted by 1 and 1.5 nm using excitation wavelengths of 280 and 295 nm, respectively (Fig. 4*C*, *insets*). A *K_d_*, _app_ of 2.9 ± 0.2 mg ml^−1^ polyG of 6–8 kDa (equivalent to 363–483 μm) was determined by fitting a one-site binding model to the intensity decrease (Fig. 4*C*). The presence of polyG did not affect *T_m_* of *Bcel*PL6 as shown by DSC (Fig. 4*D*).

### Biochemical characterization

**Figure 5.**
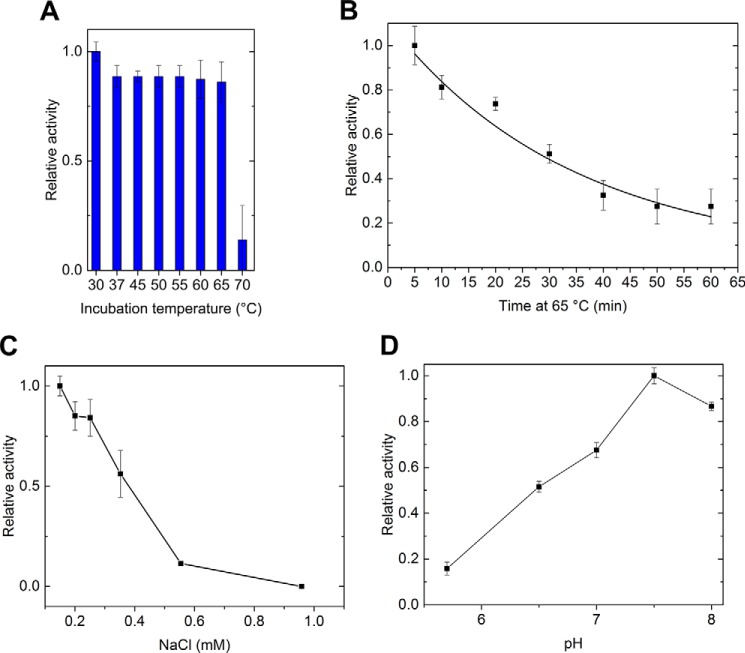
**Biochemical characterization of *Bcel*PL6.** *A*, relative activity toward alginate of *Bcel*PL6 at 30 °C after a 5-min incubation at the given temperature. *B,* inactivation of *Bcel*PL6 at 65 °C. *C,* effect of sodium chloride on *Bcel*PL6 activity toward alginate. *D,* pH activity profile of *Bcel*PL6 degradation of alginate.

*Bcel*PL6 retained full activity for alginate after 5 min at 65 °C, but lost 86% activity at 70 °C (Fig. 5*A*) in agreement with a *T_m_* of 68.8 °C (Fig. 4*D*). The inactivation of *Bcel*PL6 at 65.0 °C showed a half-life of 34 min (Fig. 5*B*). Activity toward alginate was suppressed to 50% in the presence of 350 mm NaCl and was almost completely lost in 0.95 m NaCl (Fig. 5*C*). Various acidic compounds and neutral sugars did not significantly change the activity, except for sodium-citrate presumably chelating the essential Ca^2+^ in *Bcel*PL6 leading to loss of activity (Fig. S5). The activity optimum for alginate degradation was found to be around pH 7.5 in 50 mm sodium phosphate (Fig. 5*D*).

### Three-dimensional structure

**Figure 6.**
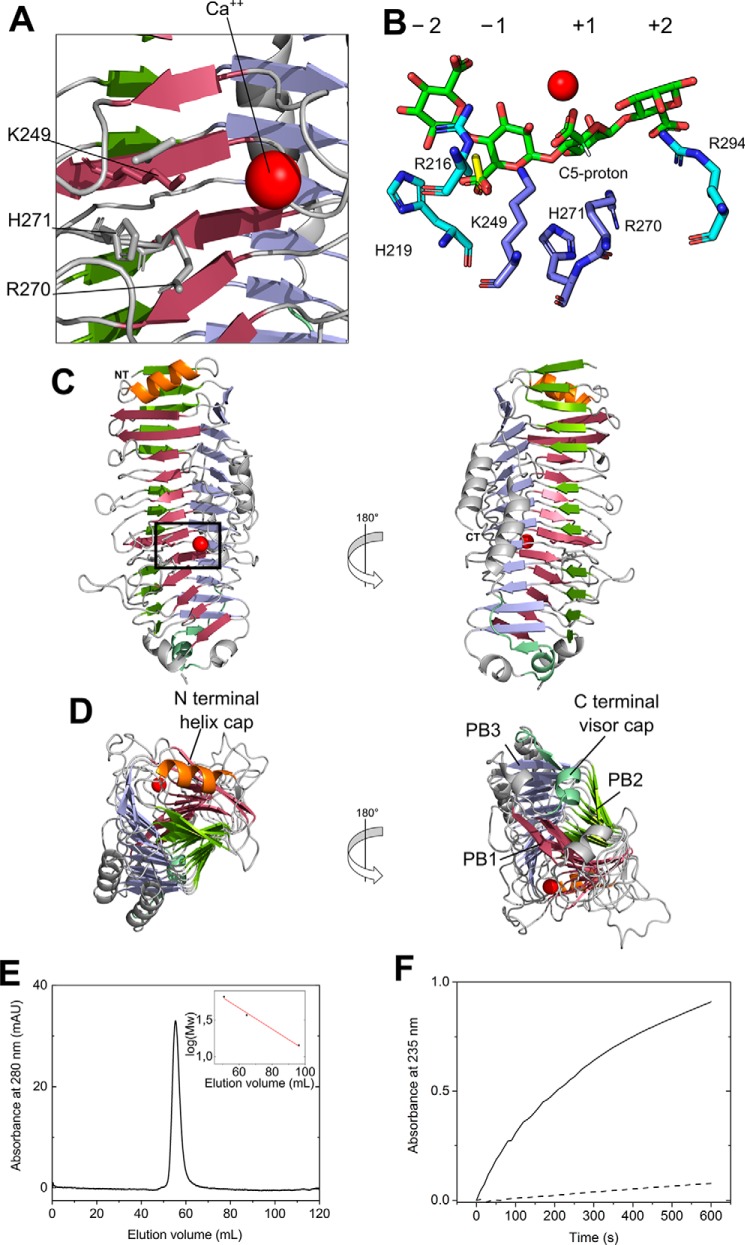
**Ribbon representation of *Bcel*PL6 (PDB 6QPS).** *A,* zoom-in of active site with PL6 conserved catalytic residues Lys-249 and Arg-270 as well as the His-271, situated between subsites −1 and +1, and the neutralizing Ca^2+^ (*red*). *B,* docked DP4M with subsites indicated. The *yellow* molecule is an acetate found in the crystal structure presumably from the crystallization solvent. *C,* overall structure of *Bcel*PL6; *black box* indicates the active-site zoom-in in *A. D,* N- and C-terminal parts of the β-helix with the capping features and sheets (*PB1–PB3*) named. *E,* analytical SEC of *Bcel*PL6 (Superdex 75). The *inset* is the standard curve of lysozyme, β-lactoglobulin, and BSA yielding a molecular mass of *Bcel*PL6 of 52.3 kDa (theoretical 52.9 kDa). *F,* increase in absorbance at 235 nm as a function of time of 4 mg ml^−1^ alginate degradation by 100 nm *Bcel*PL6 dialyzed against 50 mm HEPES, pH 7.3, 150 mm NaCl (*solid line*), or 50 mm HEPES, pH 7.3, 150 mm NaCl, 1 mm EDTA (*dashed line*).

**Figure 7.**
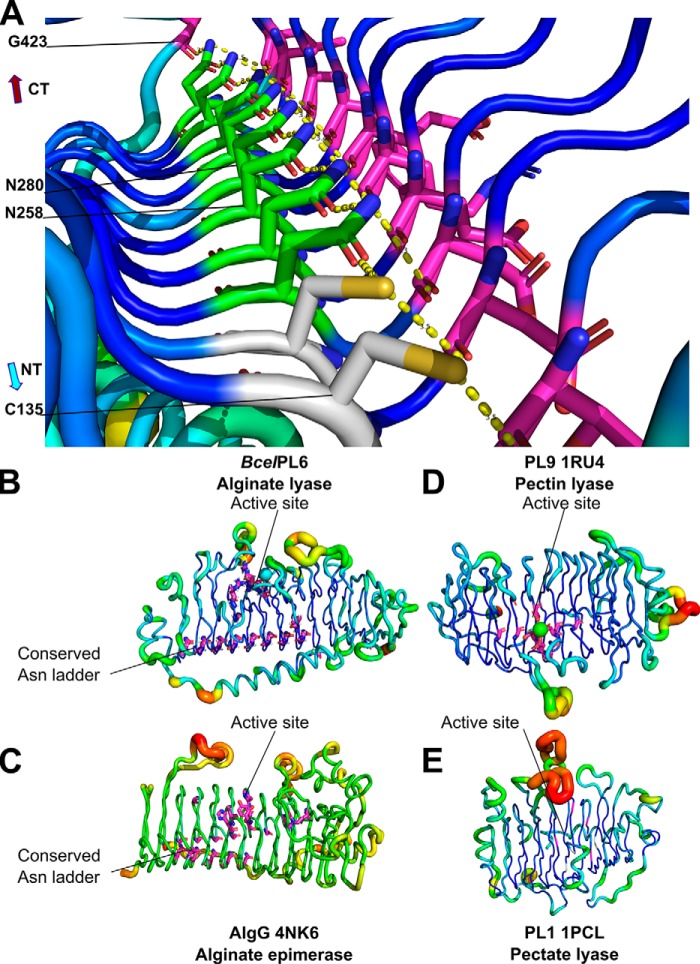
**Asparagine ladder of the T2 loops in *Bcel*PL6 and B-factor putty representation of β-helix enzymes related to *Bcel*PL6.** *A,* hydrophobic core of *Bcel*PL6 with the conserved asparagine ladder as *stick* models showing Asn (*green*) and Cys (*gray*); hydrogen bond partners (*purple*); the *broken line* (*yellow*) represents the hydrogen bond between donor and acceptor. Conserved residues (*purple*) in *B, Bcel*PL6. *C*, AlgG alginate epimerase. *D,* PL9 pectin lyase. *E,* PL1 pectate lyase.

*Bcel*PL6 crystallized from 0.2 m calcium acetate, 0.1 m Tris-HCl, pH 7.0, 20% PEG 3000 in the space group P2_1_, and the structure was solved at 1.3 Å resolution (Fig. 6; Table 2) with two molecules in the asymmetric unit. Both PISA analysis (33) and SEC (Fig. 6*E*) indicated that *Bcel*PL6 is a monomer in solution. *Bcel*PL6 is a right-handed parallel β-helix formed by three β-sheets similar to the other three available PL6 structures (PDB codes 1OFL, 5GKD, and 5Z9T) (Fig. 6*C*) (27–29). In the β-helix fold nomenclature, PB1–3 are the parallel β-sheets, and T1–3 are the connecting loops (34). A complete “helix turn” thus comprises PB1–T1–PB2–T2–PB3–T3, with PB1 being designated to contain the active site (Fig. 6*D*) (35). Although the T2 loops have a distinct shape (Fig. 7*A*, see also below), T loops generally lack secondary structure except for a T1 forming a two-turn α-helix loop in the C-terminal part of the β-helix (Fig. 6*D*). The C-terminal segment of the polypeptide (residues 432–468) adopts two three-turn α-helices parallel to the plane of β-sheet PB3 (Fig. 6*C*). The N-terminal part of the β-helix has an α-helix in the plane of PB1 (Fig. 6*D*), and the C-terminal part has an α-helix and a β-strand almost perpendicular to PB3 (Fig. 6, *C* and *D*). These structural elements match β-helix features referred to as the N-terminal helix cap and C-terminal visor cap, generally needed to avoid oligomerization and amyloid formation of β-helix proteins (36). The visor cap is kept in place by a hydrogen bond from Gly-423 to an asparagine ladder that creates an extensive hydrogen bond network on the hydrophobic side of T2 loops leading to their distinct shape (Fig. 7*A*). The asparagine ladder in *Bcel*PL6 contains 10 > 95% conserved residues across 1944 sequences sharing no more than 80% identity pairwise: Cys-135, Cys-169, **Asn-199**, **Asn-234**, **Asn-258**, **Asn-280**, **Asn-305**, **Asn-350**, **Asn-376,** and Asn-402; 7 of which (boldface) are >98% conserved (Fig. 7; Table S2).

**Table 2:** **Data collection and refinement statistics of *Bcel*PL6**

Resolution range	55.4–1.29 (1.33–1.29)
Space group	P2_1_
Unit cell	
*a* (Å)	58.62
*b* (Å)	129.95
*c* (Å)	66.99
β (°)	113.9
Wavelength (Å)	0.873
Total reflections	1,513,764 (136,645)
Unique reflections	230,084 (22,263)
Multiplicity	6.6 (6.1)
Completeness (%)	99.7 (96.6)
Mean *I*/σ(*I*)	7.23 (1.69)
Wilson *B*-factor	11.14
*R*-merge	0.123 (0.753)
*R*-meas	0.134 (0.824)
*R*-pim	0.052 (0.329)
*CC*|n=	0.998 (0.535)
*CC^a^*	0.999 (0.835)
Reflections used in refinement	230,056 (22,261)
Reflections used for *R*-free	11301 (1106)
*R*-work	0.159 (0.261)
*R*-free	0.175 (0.278)
*CC*(work)	0.976 (0.808)
*CC*(free)	0.970 (0.790)
No. of nonhydrogen atoms	8671
Macromolecules	7389
Ligands	18
Solvent	1264
Protein residues	890
Root mean square (bonds)	0.011
Root mean square (angles)	1.09
Ramachandran favored (%)	98.08
Ramachandran allowed (%)	1.92
Ramachandran outliers (%)	0.00
Rotamer outliers (%)	0.13
Clashscore	5.79
Average *B*-factor	16.81
Macromolecules	14.62
Ligands	23.27
Solvent	29.55
No. of TLS groups	12

*^a^* Numbers in parentheses refer to data in the highest-resolution shell.

Electron density indicated Ca^2+^ and two acetate molecules near the putative catalytic lysine and arginine residues in *Bcel*PL6 (Fig. S6; Table S2). The Ca^2+^ is assumed to neutralize the C6 carboxylate group at subsite +1 (Fig. 6*B*), thus facilitating substrate binding and lowering the p*K_a_* of the C5 departing proton in the lyase reaction (27, 29). This is consistent with the loss of *Bcel*PL6 activity after dialysis against EDTA (Fig. 6*F*).

### Docking of tetra-mannuronic acid (DP4M)

Attempts to solve the structure of *Bcel*PL6 in complex with DP3G, DP4G, DP3M, DP4M, or *Bcel*PL6 reaction products were unsuccessful. Therefore, DP4M was docked into the active site. Using Glide (Schrödinger suite 2016-1), 12 among the 68 docked ligand conformations had a glide score < −9 kcal mol^−1^, but in only one was the C5 proton on the side of the sugar ring that allows *syn*-elimination and points toward the catalytic residues (Fig. 6*B*). The carboxylic acid group of DP4M at subsite −1 aligns with acetate shown in the native *Bcel*PL6 structure (Fig. 6*B*) and interacts with His-219 at subsite −1, whereas C5 carboxyl groups at subsites +2 and −2 interacted with the 98% conserved Arg-294 and Arg-216, respectively (Fig. 6*B*).

**Figure 8.**
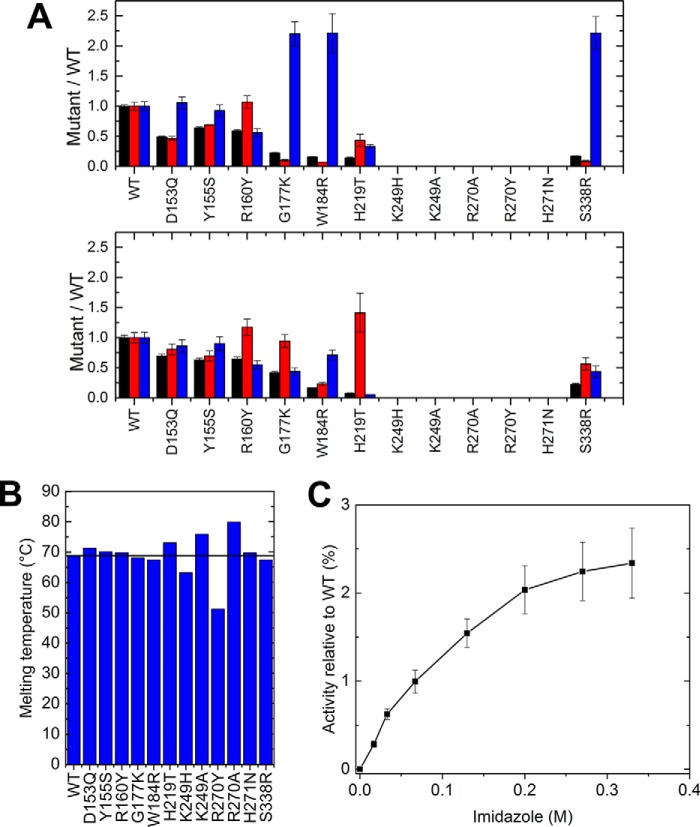
**Characterization of *Bcel*PL6 mutant enzymes.** *A,* kinetic data of WT and mutants normalized to WT values for *A*: *top,* alginate; *bottom,* polyM. *k*_cat_ (*black*), *K_m_* (*red*), and *k*_cat_/*K_m_* (*blue*). *B*, melting temperature of WT and mutants determined by DSC. The *black line* represents WT melting temperature. *C*, imidazole rescue of activity of *Bcel*PL6 H271N toward 4 mg ml^−1^ alginate.

The putative *Bcel*PL6 catalysts Lys-249 and Arg-270 were mutated to Ala or His and Ala or Tyr, respectively, where His and Tyr represent the pair of catalytic residues found in all other alginate lyases, but not in PL6 (21). These four *Bcel*PL6 mutants were inactive. Moreover, the *T_m_* of K249H and R270Y decreased by 5 and 15 °C, respectively (Fig. 8, *A* and *B*), indicating an effect on the conformational stability, whereas the corresponding alanine mutants were stabilized by several degrees (Fig. 8*B*). The 95% conserved His-271 situated between subsites +1 and −1 appears critical for activity as *Bcel*PL6 H271N was inactive, although it retained the *T_m_* of *Bcel*PL6 WT (Fig. 8*B*). Addition of imidazole restored up to 2.5% of the WT activity (Fig. 8*C*), supporting that His-271 is implicated in the function.

Alignment of *Bcel*PL6 with the crystal structures of AlyGC (PDB 5GKD) and AlyF (PDB code 5Z9T) highlighted conserved residues important for catalytic activity and previously investigated by mutational analysis (27, 28). However, *Bcel*PL6 differs by Arg-160 that corresponds to AlyGC Tyr-130 and Trp-172 in AlyF (Table S3) as well as two notable positions at subsites +1 and −1, where *Bcel*PL6 has His-219 and Ser-338 corresponding to AlyGC T190 and Arg-303, respectively, both situated to interact with substrate. Notably, His-219 binds with the docked DP4M at subsite −1 (Fig. 6*B*).

**Figure 9.**
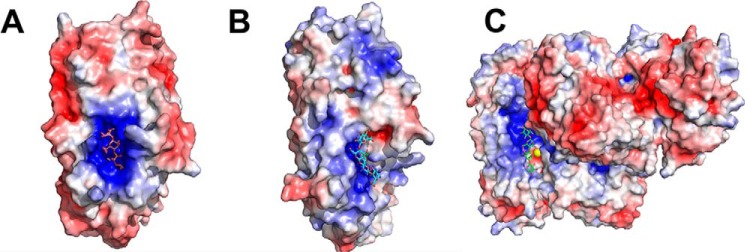
**Electrostatic surface representation of PL6 alginate lyases.** *A,* AlyF in complex with DP4G (PDB code 6ITG). *B, Bcel*PL6 in complex with DP4M docked into the active site. *C,* AlyGC in complex with DP4M (PDB code 5GKQ). Figure was prepared using PyMOL 2.0 and APBS electrostatics.

**Figure 10.**
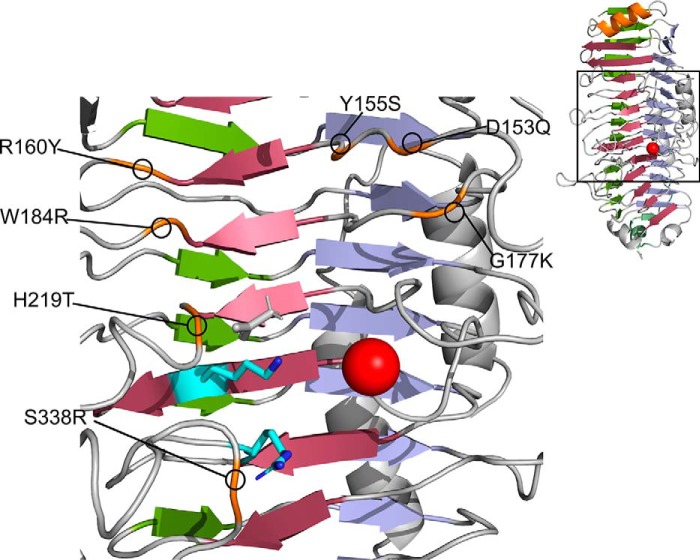
**Positions mutated in the *Bcel*PL6 structure to match corresponding residues in AlyGC (PDB code 5GKD).**

The large positively-charged active-site area in both AlyF and AlyGC did not resemble *Bcel*PL6 (Fig. 9). Introducing positive and other side chains by mutation in *Bcel*PL6 to mimic AlyGC (Fig. 10) did not result in activity on polyG, the substrate preferred by both AlyGC and AlyF. For polyM and alginate, *Bcel*PL6 D153Q and Y155S had decreased *k*_cat_ and *K_m_* values (Fig. 8*A*), whereas R160Y showed lower *k*_cat_ and unaltered *K_m_* values for these two substrates. Furthermore, W184R has 6-fold reduced *k*_cat_ on both polyM and alginate, and 4- and 15-fold reduced *K_m_* on polyM and alginate, respectively (Fig. 8). These residues thus play a role in activity but appear not to contribute to controlling the polyM *versus* polyG specificity. Surprisingly, G177K, H219T, and S338R had *K_m_* values decreased by 3–12-fold on alginate, but practically unchanged on polyM compared with WT. The *k*_cat_ was lowered by 5–10-fold on both substrates (Fig. 8*A*).

**Figure 11.**
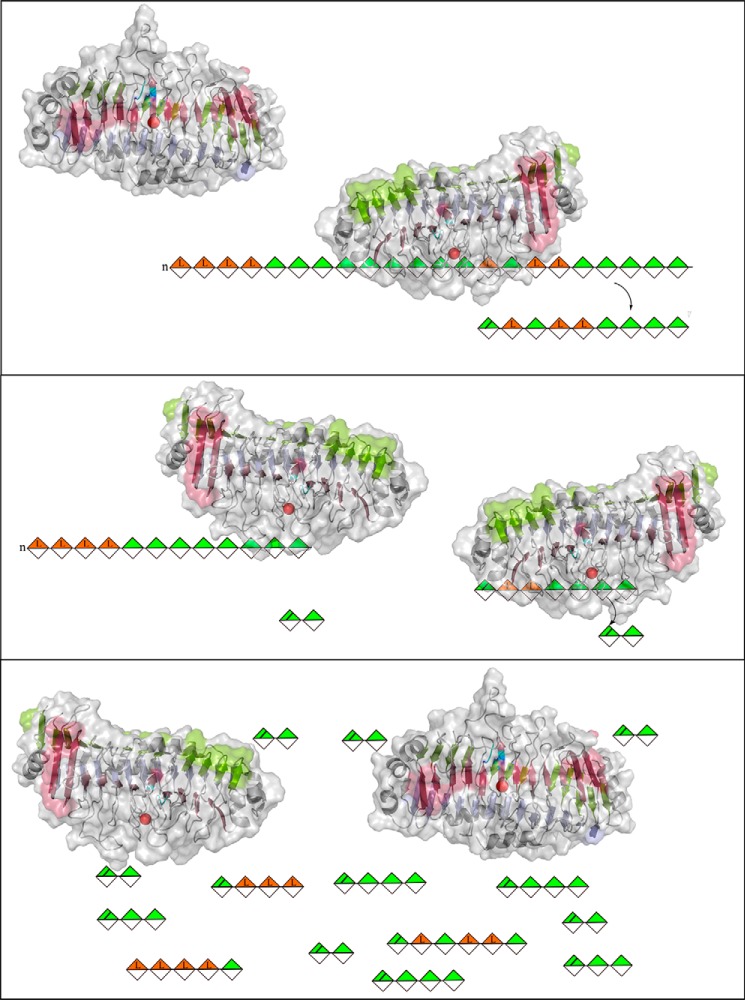
**Cartoon of product release manner for *Bcel*PL6, 1,4-β-d-mannuronic acid (*green*), and 1,4-α-l-guluronic acid (*orange*).** *Bcel*PL6 binds to alginate and cleaves off a larger unsaturated oligosaccharide (up to DP7) (*top*). If the oligosaccharide is ≥DP4 and contains an M–M linkage, it can be cleaved again to yield DP2 (*middle*). This process continues until the final product mixture is achieved (*bottom*).

## Discussion

*Bcel*PL6 is the first structure-determined and thoroughly-characterized PL6 alginate lyase from HGM. In PL6, sequence-based prediction of substrate specificity is generally difficult and seems to vary with small structural differences at the active site (27). The present results on *Bcel*PL6 therefore strengthen future prediction of substrate specificity and mode of action of related enzymes. An extremely low activity on polyG and polyMG distinguishes *Bcel*PL6 from previously characterized PL6 alginate lyases, which were found to be polyMG (20, 23) and or polyG-specific (20, 24, 27). A polyM preference was so far seen only for PL6 from *Pseudomonas* sp. PapOS ALG-9, which also showed ∼25% activity on polyG (37). Alginate lyases preferring polyM similarly to *Bcel*PL6 have been identified from PL5, -7, -14, -15, -17, -18, -32, and -36 (17–19, 38–42).

Alginate endo-lyases are described to release unsaturated oligosaccharides, which are then further degraded (2). Exo-lyases by contrast release unsaturated monouronic acid (21, 22). *Bcel*PL6 probably requires the reducing end for further degradation of initially produced oligosaccharides (Fig. 11), because octa-mannuronic acid was a substrate, whereas the corresponding alcohol, prepared by reduction with NaBH_4_, was not degraded. Previously, some PL8 chondroitin lyases were reported to act from the substrate-reducing end (43).

### BcelPL6 structure

The two other PL6 alginate lyase structures, AlyGC from the marine bacterium *Paraglaciecola chatamensis* S18K6T (PDB code 5GKD) and AlyF *V. splendidus* OU2 (PDB code 5Z9T), are a solution homodimer (27) and a monomer, respectively (28). *Bcel*PL6 is also monomeric (Fig. 6). The activity of polyG-specific AlyGC (27), the polyM-specific *Bcel*PL6, and the chondroitin B lyase (PDB code 1OFL) (29) depends on Ca^2+^. This, however, was reported not to be the case for the polyG-specific AlyF (28).

*Bcel*PL6 has a rather flat and open active site; in AlyGC the C-terminal dimerization domain partly covers the active site forming a cleft (27), and in AlyF, loops form a closed active site over the nonreducing end of the bound DP4G (28). This difference in active-site topology may explain that *Bcel*PL6 releases oligosaccharides and AlyGC monosaccharides (Fig. 9). As *Bcel*PL6 is a single-domain monomer, the subunit rotation between the two domains of AlyGC suggested to shape the cleft involved in catalysis (22, 27) cannot be a general feature of PL6. Also, *Bcel*PL6 has a notably lower salt tolerance than the marine AlyGC, which remains 50% active in 0.5 m NaCl (27) as opposed to *Bcel*PL6 that only retains 10% activity at 0.55 m NaCl, reflecting adaptation to their individual niches.

The docked DP4M interacts with *Bcel*PL6 Arg-294 and Arg-216 (Fig. 6*B*), and the corresponding arginines in AlyGC and AlyF were shown to be important for activity (27, 28). Notably, when the C5 proton is in a position compatible with proton abstraction by the catalytic base, the direction of the orientation of DP4M in the complex was flipped compared with DP4M bound in AlyGC (PDB 5GKQ) (Fig. 9; Fig. S7). This fits well with *Bcel*PL6 requiring the reducing end for degrading oligosaccharides (Fig. S2*D*). It has been suggested that in the *syn*-acting M-specific alginate lyase A1-III from PL5, a single tyrosine acts as both catalytic base and acid (41). In *Bcel*PL6, however, the geometry of the docked DP4M complex, combined with mutational analysis of Lys-249, Arg-270, and His-271 (Fig. 8), does not allow for accurate determination of whether there is one or more catalytic residues.

### Structure–activity relationships

The high preference of *Bcel*PL6 for polyM matches with the monomeric structure and its flat active site (Fig. 9), as M-specific enzymes apply a *syn*-mechanism (21, 22) that requires both of the catalytic functions to be on the same side of the sugar ring (see Figs. 1 and 6, *A* and *B*). Still, PL6 enzymes with dual M and G specificity have been reported, which indicates that structure-based explanation of the specificity needs to be further developed (23, 24, 27). Conservation of His-271 at the active site and loss of activity of *Bcel*PL6 H271N suggest this residue is critical for activity (Figs. 6 and 8 and Table S2). However, the effect of mutating His-271 in the G-specific PL6 lyases AlyGC and AlyF is negligible (27, 28), and the equivalent chondroitinase B mutant retains 25% activity toward dermatan sulfate (44). Taken together, it indicates that this histidine plays a particularly important role in the depolymerization of polyM. Notably, the mannuronate epimerases AlgG and AlgE4, which also have a parallel β-helix fold, both have a conserved histidine in the active site (45, 46). This residue is proposed to act as catalytic base abstracting the C5 proton at subsite +1 as part of the epimerization mechanism with an arginine functioning as charge neutralizer (45, 46). In both of these two available epimerase structures, the distance between histidine and arginine is about the same as in *Bcel*PL6 (45, 46). There could be an evolutionary relationship between alginate epimerases and lyases, and His-271 may function as a catalytic base in the M-specific *Bcel*PL6. PROpKa (47) calculates the p*K_a_* value of His-271 to 2.0 without ligand and −0.17 in the docked complex with DP4M. Therefore, His-271 cannot become protonated at pH 7.3. Hence, His-271 is likely not active as a catalytic group, although it may still be critical for substrate recognition for polyM as supported by the loss of activity of *Bcel*PL6 H271N.

### PolyG as activator

In the presence of polyG, *K_m_* and *k*_cat_ both increased by about the same factor as for *Bcel*PL6 acting on alginate (Fig. 3*B*); thus, the catalytic efficiency was not affected by polyG. Assuming the reaction follows the simple mechanism shown in Reaction 1,

$$\begin{array}{c} E+\text{S}\overset{k_{f1}}{\underset{k_{r1}}{⇌}}\text{ES}\overset{k_{f2}}{\underset{k_{r2}}{⇌}}E+P\\ \text{Reaction}\;1 \end{array}$$

where *k* denotes a reaction rate then we achieve Equations 1 and 2,

(Eq. 1) $$K_{m}=\frac{k_{r1}+k_{f2}}{k_{f1}}$$

(Eq. 2) $$k_{f2}=k_{\text{cat}}$$

*K_m_* and *k*_cat_ both increase if *k_f_*_2_ increases. If *k_f_*_1_ increases, *K_m_* would decrease, whereas *k*_cat_ remains unaltered. Therefore, it may be *k_f_*_2_ that is affected by addition of polyG. One possibility is that polyG binds stronger to the active site than the products but weaker than the substrates, thus being able to expel the reaction product, which is easily displaced by substrate. This is supported by *K_m_* = 0.58 ± 0.04 mg ml^−1^ for alginate and *K_d_*, _app_ = 2.9 ± 0.2 mg ml^−1^ for polyG binding to *Bcel*PL6. That *k*_cat_/*K_m_* is unchanged by the addition of polyG further supports that it is the later steps of the reaction that are affected.

### Asparagine ladder

The five polysaccharide lyase families PL1, -3, -6, -9, and -16 (26, 48–51) adopt a parallel β-helix catalytic domain fold. Among these, PL1, -6, and -9 contain a so-called asparagine ladder (26, 49, 51). The 10-“step” asparagine ladder in *Bcel*PL6 is the longest reported to date and spans almost the entire length of the β-helix (10 of 12 turns, Fig. 7). Remarkably, in PL6 a large number of the asparagine residues constituting the ladder are essentially invariant (Table S2) suggesting they are vital for folding and stability of the PL6 β-helix. The hydrogen-bonding network of the asparagine ladder in *Bcel*PL6 ends by Gly-423 in the C-terminal visor cap, indicating its possible implication in prevention of amyloid fibril formation (36). Sequence analysis of families PL1, PL9, and AlgG epimerases revealed that conserved asparagine ladders are only found in β-helix enzymes acting on alginate and not in pectin and pectate lyases (Fig. 7; Table S2). Asparagine ladders may confer rigidity and prevent conformational rearrangement of the secondary structure upon binding of the polyelectrolyte alginate.

In summary, recombinant *Bcel*PL6 from *B. cellulosilyticus* is M-specific and produces mainly disaccharides as end products from alginate and di- and trisaccharides from polyM. *Bcel*PL6 does not degrade polyG, polyMG, or acetylated polyM to a significant degree. The *Bcel*PL6 crystal structure solved to 1.3 Å is monomeric similarly to AlyF and opposed to the homodimeric PL6 alginate lyase AlyGC. The more positively charged and narrow active site in AlyGC compared with *Bcel*PL6 may explain the specificity difference. Moreover, the monomeric *Bcel*PL6 only allows *syn*-elimination making it mannuronate-specific. The conserved His-271 at subsite +1 was found to be crucial for activity. The present thorough characterization of *Bcel*PL6 improves the general insight into PL6 structure and function and will advance future identification and specificity assignment of alginate lyases from this family.

## Experimental procedures

### Materials

*B. cellulosilyticus* CRE21 was purchased from Deutsche Sammlung von Mikroorganismen und Zellkulturen DSM number 14838 (Germany). Brain Heart Infusion broth and LB broth were purchased from Sigma. Alginate *M̄_n_* of M/G ratio = 0.6 was a kind gift of DuPont Nutrition and Health (Denmark). PolyM (F_G_ = 0.0, *M̄*_w_ = 3 kDa) was obtained from an epimerase-negative AlgG mutant of *Pseudomonas fluorescens* (52). PolyG *M̄*_w_ = 6–8 kDa (F_G_ = 0.97) was prepared as described previously (2); alternating polyMG (F_G_ = 0.46, F_GG_ = 0.0) of DP30 was made by epimerization of polyM *in vitro* using AlgE4 (53). Alginate oligomers for product identification were obtained by fractionation of alginate hydrolysates on SEC columns as described previously (2). Octa-mannuronic acid (DP8M) was reduced by NaBH_4_ opening the ring structure at the reducing end without affecting the remaining residues (54, 55). Briefly, to DP8M in MQ water (4 mg ml^-1^) was added NaBH_4_ (s) 5% (w/v). Solid substances added to aqueous substances are denoted (s) and (aq), respectively. After 1 h at ambient temperature, the mixture was kept on ice, and glacial acetic acid was added dropwise until no further gas production was observed, and pH was adjusted to 7.0 with dilute NaOH. The sample was dialyzed against two shifts of 50 mm NaCl and then against MQ water until conductivity was <2 μS, followed by freeze drying.

### Bioinformatics

Lyase sequences were retrieved from the Uniprot database (56) using protein blast with 1PCL, 1RU4, and 4NK6 and *Bcel*PL6 as queries, and clustered on a 90% identity threshold on CD-Hit (57). Iterative multiple sequence alignments were performed with Clustal Omega (58) to select the maximum number of sequences of 15–80% pairwise identity. The degree of amino acid residue conservation was assessed on the examined sequences.

### Growth of B. cellulosilyticus

Isolation of genomic DNA. *B. cellulosilyticus* was grown overnight under anaerobic conditions (Whitley DG250 anaerobic work station) in 5 ml of Brain Heart Infusion medium supplemented with 5 μg ml^−1^ hemin at 37 °C. Cells were harvested by centrifugation (2800 × *g*, 4 °C, 20 min; Eppendorf 5810 R centrifuge), and genomic DNA was isolated (59). DNA concentration was determined spectrophotometrically at 260 nm.

### PCR, cloning, and mutagenesis

PCR on genomic DNA was performed using Phusion High-Fidelity DNA Polymerase (New England Biolabs) with the following primers for In-Fusion cloning: 5′-CGCGCGGCAGCCATATGAAAGAGTATACATTTTCACCGAAAG-3′ and 5′-GCTCGAATTCGGATCCTCAGCGATTCGTATCGATATGG-3′ covering the *Bcel*PL6 gene from residue 19 to omit the signal peptide predicted by SignalP (60). The PCR product and the p28a+ plasmid linearized by BamHI and NdeI (New England Biolabs) were purified by agarose gel electrophoresis and ligated using the In-Fusion cloning kit (Takara Bio) according to the manufacturer's protocol. The resulting plasmid was verified by sequencing (GATC Biotech, Germany). This construct N-terminally extends recombinant *Bcel*PL6 by MGSSHHHHHHSSGLPRGSH (a His-tag and a thrombin cleavage site). The plasmid was transformed in *Escherichia coli* BL21. Site-directed mutagenesis was performed using a QuickChange lightning site-directed mutagenesis kit (Agilent), and the point mutation was verified by sequencing (GATC Biotech, Germany). Mutagenesis primers can be found in Table S4.

### Production and purification of recombinant BcelPL6

The cryostock was cultured in LB medium at 37 °C overnight, inoculated (10 ml) in 1 L LB-KAN medium, and grown to OD_600_ = 0.6–0.8 (37 °C, 160 rpm). Expression was induced by isopropyl β-d-thiogalactopyranoside added to 0.5 mm followed by incubation (22 °C, 16 h). Cells were harvested by centrifugation (5000 × *g*, 4 °C, 15 min) and stored at −20 °C. Pellet corresponding to 0.33 liters of culture was resuspended in 20 ml of 50 mm HEPES, pH 7.3, 150 mm NaCl, lysed (pressure cell homogenizer; Stansted Fluid Power, UK), and centrifuged (20,000 × *g*, 20 min). HisPur^TM^ nickel-nitrilotriacetic acid resin (2 ml; Thermo Fisher Scientific), pre-equilibrated in 20 ml of 50 mm HEPES, pH 7.3, 150 mm NaCl, was added to the supernatant with gentle mixing (30 min). The resin was washed with 20 ml of 20 mm imidazole, 50 mm HEPES, pH 7.3, 150 mm NaCl, and the protein was eluted by 10 ml of 300 mm imidazole in the same buffer. Eluate (10 ml) was immediately gel-filtered (Hi-load Superdex 75 26/60; GE Healthcare) in 50 mm HEPES, pH 7.3, 150 mm NaCl at a flow rate of 2 ml min^−1^. Protein purity was assessed by SDS-PAGE (Fig. S1), and the concentration was determined spectrophotometrically at 280 nm using the predicted (Protparam) ϵ = 65,820 m^−1^ cm^−1^. The yield was typically about 30 mg liter^−1^ culture. All purification steps were performed at 4 °C.

### Enzyme activity and kinetics

Substrates (5 mg ml^−1^) dissolved in 50 mm HEPES, pH 7.3, 150 mm NaCl were centrifuged prior to use. Kinetics were determined for 100 nm *Bcel*PL6 on alginate and polyMG, 50 nm on polyM, 50 nm as well as 6 μm on polyG using 0–4 mg ml^−1^ substrates. Samples were mixed in a 96-well UV-star chimney well plate (In Vitro, Australia), equilibrated at 37 °C (5 min), and enzyme was added. Formation of unsaturated uronic acid products was measured spectrophotometrically at 235 nm every 10 s for 10 min at 37 °C (Bio-Tek Powerwave XS; Holm and Halby, Denmark) (16, 61) and converted to molar concentration using ϵ = 6150 m^−1^ cm^−1^ (62, 63). The initial part of progress curves was analyzed by linear regression (Origin 2016; Originlab), and initial rates *versus* substrate concentrations were fitted to the Michaelis-Menten model (64). All data points are with the standard deviation of a triplicate. Activation by 0–3.33 mg ml^−1^ polyG was assayed either at 3 mg ml^−1^ alginate for 50 nm *Bcel*PL6 or in kinetic assays (as above) at 0, 0.16, 0.66, and 3.33 mg ml^−1^ polyG. PolyG was thoroughly dialyzed to remove Ca^2+^ to avoid an artifact by reconstituting eventually Ca^2+^-depleted *Bcel*PL6. Activation and inhibition by sodium citrate sodium phosphate, sodium carbonate, maltotriose, lactose, and sodium acetate were assayed as described above at a compound concentration of 2 mg ml^−1^.

### Spectrofluorometry of polyG binding

*Bcel*PL6 (500 nm) was emission scanned with and without polyG at 320–365 nm with excitation at 280 or 295 nm and also analyzed by measuring polyG (0–4.5 mg ml^−1^) elicited decrease in fluorescence intensity at 340 nm with excitation at 280 nm (LS-55 luminescence spectrometer; PerkinElmer Life Sciences). The *K_d_*_, app_ was obtained from the binding curve that was fitted to a standard one site-binding model as shown in Equation 3,

(Eq. 3) $$y=y_{0}+\frac{a\left[\text{L}\right]}{K_{d}+\left[\text{L}\right]}$$

where [L] is ligand concentration; *y*_0_ is fluorescence intensity at [L] = 0; and *y*_0_ + is fluorescence intensity at saturation. This model assumes that [L]_free_ ∼ [L]_total_, which is only valid for weak interactions.

### Product analysis by size-exclusion chromatography and MS

Mixtures (2 ml) of 4 mg ml^−1^ alginate and 100 nm *Bcel*PL6 were incubated at 37 °C and inactivated at 90 °C at 0, 1, 5, 10, 20, 30, 60, 90, and 120 min, and the reaction products were separated by SEC (Superdex 200 16/60) in 50 mm HEPES, pH 7.3, 700 mm NaCl at a flow rate of 0.5 ml min^−1^ and monitored at 235 nm. Breakdown for 20 mg ml^−1^ alginate by 100 nm *Bcel*PL6 was analyzed at 0, 2, 5, 10, 30, 60, 120, and 240 min by LC-ESI-MS. Collected samples were stored at −20 °C until quantification of oligosaccharide products by LC-ESI-MS (Amazon SL iontrap; Bruker Daltonics, Germany, coupled to UltiMate 3000 UHPLC equipped with an Ultimate RS diode array detector (235 nm), Dionex). Samples (5 μl) in 50% ACN were injected (GlycanPac AXH-1 column, 150 × 2.1 mm; Thermo Fisher Scientific, Waltham, MA) and eluted at 0.4 ml min^−1^ at 30 °C by a three-eluent system of water (solvent A), 100 mm ammonium formate pH 5 (solvent B), and ACN (solvent C), keeping 19% A at time (in minutes) with the eluent profile: 0–10 isocratic 1% B; 10–45 linear gradient to 19% B; 45–50 linear gradient to 1% B; 50–60 isocratic 1% B. The electrospray was operated in negative mode with enhanced resolution mode and scan range 100–2000 *m*/*z*, smart parameter setting of 500 *m*/*z*, capillary voltage at 4.5 kV, end plate off-set 0.5 kV, nebulizer pressure at 3.0 bars, dry gas flow at 12.0 liters min^−1^, and dry gas temperature at 280 °C. Identification by *m*/*z* and quantification at 235 nm using ϵ = 6150 m^−1^ cm^−1^ (62, 63) was done in Compass QuantAnalysis 2.2 (Bruker Daltonics, Germany) using Equation 4,

(Eq. 4) $$c=\frac{\text{dilution}\times \text{area}\times \text{flow}}{\text{length}\times \text{ext}.\;\text{coeff}.\times \text{injection}\;\text{volume}}$$

ESI-MS results were confirmed by MALDI-TOF for 0- and 120-min reaction mixtures (buffer exchanged to 300 mm acetic acid, Hiprep desalt 26/110, GE Healthcare) spotted onto an MTP Anchor chip target with 9 mg ml^−1^ DHB matrix in 30% ACN analyzed by MALDI-TOF/TOF MS (Ultraflex II, Bruker Daltonics) in linear positive mode. Mass spectra were analyzed using Flex Analysis (Bruker Daltonics).

### Differential scanning calorimetry

*Bcel*PL6 WT and mutants dialyzed against 50 mm Na_2_HPO_4_/NaH_2_PO_4_ pH 7.3, 150 mm NaCl (3 × 100-fold dilution, 10–14-kDa cutoff, 4 h, 4 °C; SpectrumLabs, Greece) were subjected at 1 mg ml^−1^ to DSC (20–90 °C; scan rate of 1 °C min^−1^) at constant pressure of 3 atm (NANO DSC; TA). Reversibility of unfolding was examined by scanning the sample twice. The reference cell contained dialysis buffer. A blank scan of dialysis buffer was subtracted as baseline and the data were converted to molar heat capacity using NanoAnalyze (TA).

### Analytical size-exclusion chromatography

*Bcel*PL6 (2 ml, 10 μm) was analyzed by SEC (Hiload Superdex 75 16/60) in 50 mm HEPES, pH 7.3, 150 mm NaCl at 4 °C (flow rate: 1 ml min^−1^). Lysozyme (14.4 kDa), β-lactoglobulin A (36.6 kDa), and BSA (66.5 kDa) were used for calibration.

### Crystallization and X-ray diffraction

*Bcel*PL6 dialyzed against 50 mm HEPES, pH 8.0, 50 mm NaCl (3 × 100-fold dilution, 4 h, 4 °C, 10–14 kDa cutoff) was concentrated to 20 mg ml^−1^ (10-kDa Amicon centrifugal filtration column; Merck) and used with MCSG-1 crystal screen kit (Anatrace) according to the manufacturer's protocol. Briefly, each reservoir in four 24-well sitting drop crystallization plates (Hampton Research) was filled (500 μl) with one of the 96 different conditions, and 2 μl of protein solution was placed on the drop shelf and mixed with 2 μl of reservoir solution prior to sealing the plate (Crystal Clear Sealing tape; Hampton Research). The plates were stored 12 days at ambient temperature, and the crystals obtained (with 0.2 m calcium acetate hydrate, 0.1 m Tris-HCl, pH 7, 20% (w/v) PEG 3000) were flash-frozen with PEG 400 as cryoprotectant, using liquid nitrogen. Diffraction data were collected at the ESRF (ID23-2; Grenoble, France).

### Data processing, structure solution, and model building

Diffraction images were processed automatically with Dials using the Xia2 interface and scaled with aimless (see Table 2 for details) (65–69). The structure of AlyGC (PDB code 5GKD) was used for molecular replacement with Phaser (70). First, AlyGC chain A (residues 2–443) was extracted and used as input to Phenix.sculptor (71) to generate the input model for molecular replacement. Phenix (72) was used with the Phaser simple component interface to run a molecular replacement searching for two molecules in the asymmetric unit as indicated by the Matthews coefficient (*V_m_* = 2.18 and solvent content of 43.6% with two molecules in the asymmetric unit). A final TFZ value of 30.2 indicated a clear solution, and after a round of automated model building with AutoBuild (73), a model with *R*_work_/*R*_free_ of 23.86/26.14 and 884 residues was produced. Model building was completed by several rounds of rebuilding in Coot (74) with refinement using Phenix.refine (75) at 1.3 Å resolution using riding hydrogens and six TLS groups per monomer. Ca^2+^ was added in the known binding site. An acetate from the crystallization conditions could be modeled in the active site. The structure was deposited to the Protein Data Bank with PDB code 6QPS.

### Glide docking of tetramannuronic acid (DP4M)

DP4M was built in Accelrys Discovery Studio. *Bcel*PL6 and DP4M were prepared using Protein Preparation Wizard and LigPrep programs, respectively, within the Schrödinger suite 2016-1 (Small-Molecule Drug Discovery Suite 2016-1, Schrödinger, LLC, New York). Protein and ligand preparation were performed at pH 7.3 (PROpka (47) and Epik (76, 77)) with default settings for the remaining parameters.

Grid generation was carried out using default parameters. The docking region was centered at the geometric center determined by the positions of Ca^2+^, Arg-216, Lys-249, and Arg-270, and the threshold of ligand size was set to 25 Å, which is ∼7 Å longer than the maximum length of DP4M. Docking experiments were performed by Glide (78) using default settings. The first experiments were conducted using Glide SP (78), and the structure with the best glide SP score was used in a Glide XP (78) run. In all energy calculations the OPLS3 force field (79) was applied.

## Author contributions

E. G. P. S. and B. S. conceptualization; E. G. P. S. and F. F. data curation; E. G. P. S., C. D. A., F. F., J. H., D. T., and G. H. P. formal analysis; E. G. P. S. and B. S. supervision; E. G. P. S., C. D. A., F. F., and D. H. W. validation; E. G. P. S., C. D. A., F. F., J. H., G. H. P., and D. H. W. investigation; E. G. P. S. and F. F. visualization; E. G. P. S., F. F., A. S., G. H. P., B. E. C., F. L. A., and D. H. W. methodology; E. G. P. S. and B. S. writing-original draft; E. G. P. S. and B. S. project administration; E. G. P. S., C. D. A., F. F., D. T., F. L. A., D. H. W., and B. S. writing-review and editing; A. S., B. E. C., F. L. A., D. H. W., and B. S. resources; D. T. and G. H. P. software; B. S. funding acquisition.

## Supplementary Material

Supporting Information

## Footnotes

3 Please note that the JBC is not responsible for the long-term archiving and maintenance of this site or any other third party hosted site.

## Footnotes

2 The abbreviations used are:

M: β-d-mannuronic acid
*Bcel*PL6: family 6 polysaccharide lyase from *B. cellulosilyticus* CRE21
Δ: 4-deoxy-l-*erythro*-hex-4-enopyranosyluronic acid
DP3G: DP4G, DP3M, DP4M, tri- or tetrasaccharides of α-l-guluronic acid or β-d-mannuronic acid
DP8M: octa-mannuronic acid
LC-ESI-MS: liquid chromatography–electron spray ionization–mass spectrometry
HGM: human gut microbiota
G: α-l-guluronic acid
*M̄_n_*: number average molecular weight
*M̄*_w_: weight average molecular weight
PDB: Protein Data Bank
PL: polysaccharide lyase
PL6: polysaccharide lyase family 6
polyG: poly-α-l-guluronic acid
polyM: poly-β-d-mannuronic acid
polyMG: alternating or random β-d-mannuronic acid and α-l-guluronic acid polymer
SEC: size-exclusion chromatography
DSC: differential scanning calorimetry
ACN: acetonitrile
PUL: polysaccharide utilization loci.
