# Supplementary material for: Structural and functional aspects of mannuronic acid–specific PL6 alginate lyase from the human gut microbe Bacteroides cellulosilyticus
Source: J Biol Chem. 2019 Sep 17;294(47):17915–30. doi: 10.1074/jbc.RA119.010206 (PMC6879350; doi:10.1074/jbc.RA119.010206)
Supplement: Supporting Information [file supp_294_47_17915__index.html]

Structural and functional aspects of mannuronic acid–specific PL6 alginate lyase from the human gut microbe Bacteroides cellulosilyticus — Structure and function of polyM specific alginate lyase — Structural and functional aspects of mannuronic acid–specific PL6 alginate lyase from the human gut microbe Bacteroides cellulosilyticus — Structure and function of polyM-specific alginate lyase — Supporting Information 

# Structural and functional aspects of mannuronic acid–specific PL6 alginate lyase from the human gut microbe *Bacteroides cellulosilyticus*

## Supporting Information

- Supporting Information (to be published online) - Purification gel, Additional specificity analysis, Additional MS analysis, compound screening for inhibition,active site electron density, conservation analysis, primer table
