# Supplementary material for: Structural and functional aspects of mannuronic acid–specific PL6 alginate lyase from the human gut microbe Bacteroides cellulosilyticus
Source: J Biol Chem. 2019 Sep 17;294(47):17915–30. doi: 10.1074/jbc.RA119.010206 (PMC6879350; doi:10.1074/jbc.RA119.010206)
Supplement: Supporting Information [file supp_RA119.010206_154323_2_supp_395559_pxx9kc.docx]

Supporting information for:

**Mannuronic acid specific PL6 alginate lyase from human gut microbiota – Structure and functional aspects**

**Emil G. P. Stender**^‡^, **Christian Dybdahl Andersen**^‡^, **Folmer Fredslund**^§^, **Jesper Holck**^‡^, **Amalie Solberg**^¶^, **David Teze**^‡^, **Günther H. J. Peters^װ^**, **Bjørn E. Christensen**^¶^, **Finn L. Aachmann**^¶^, **Ditte H. Welner**^§^, and **Birte Svensson**^‡*^

*From* ^‡^*Department of Biotechnology and Biomedicine, Technical University of Denmark, DK-2800 Kgs. Lyngby, Denmark,* ^§^*Novo Nordisk Foundation Center for Biosustainability, Technical University of Denmark, DK-2800 Kgs. Lyngby, Denmark,* ^¶^*Department of Biotechnology and Food Science, NTNU^,^ Norwegian University of Science and Technology, N-7491 Trondheim, Norway,* **^װ^***Department of Chemistry, Technical University of Denmark, DK-2800 Kgs. Lyngby, Denmark*

^*^To whom correspondence should be addressed: Department of Biotechnology and Biomedicine, Technical University of Denmark, Søltofts Plads, building 224, DK-2800 Kgs. Lyngby, Denmark. Tel.: +4545252740; E-mail: bis@bio.dtu.dk.

**Running title:** Structure and function of polyM specific alginate lyase

**Keywords:** alginate lyase, *Bacteroides cellulosilyticus* CRE21, crystal structure, parallel β-helix, asparagine ladder, molecular docking, enzyme kinetics, enzyme mechanism, mutational analysis, imidazole rescue


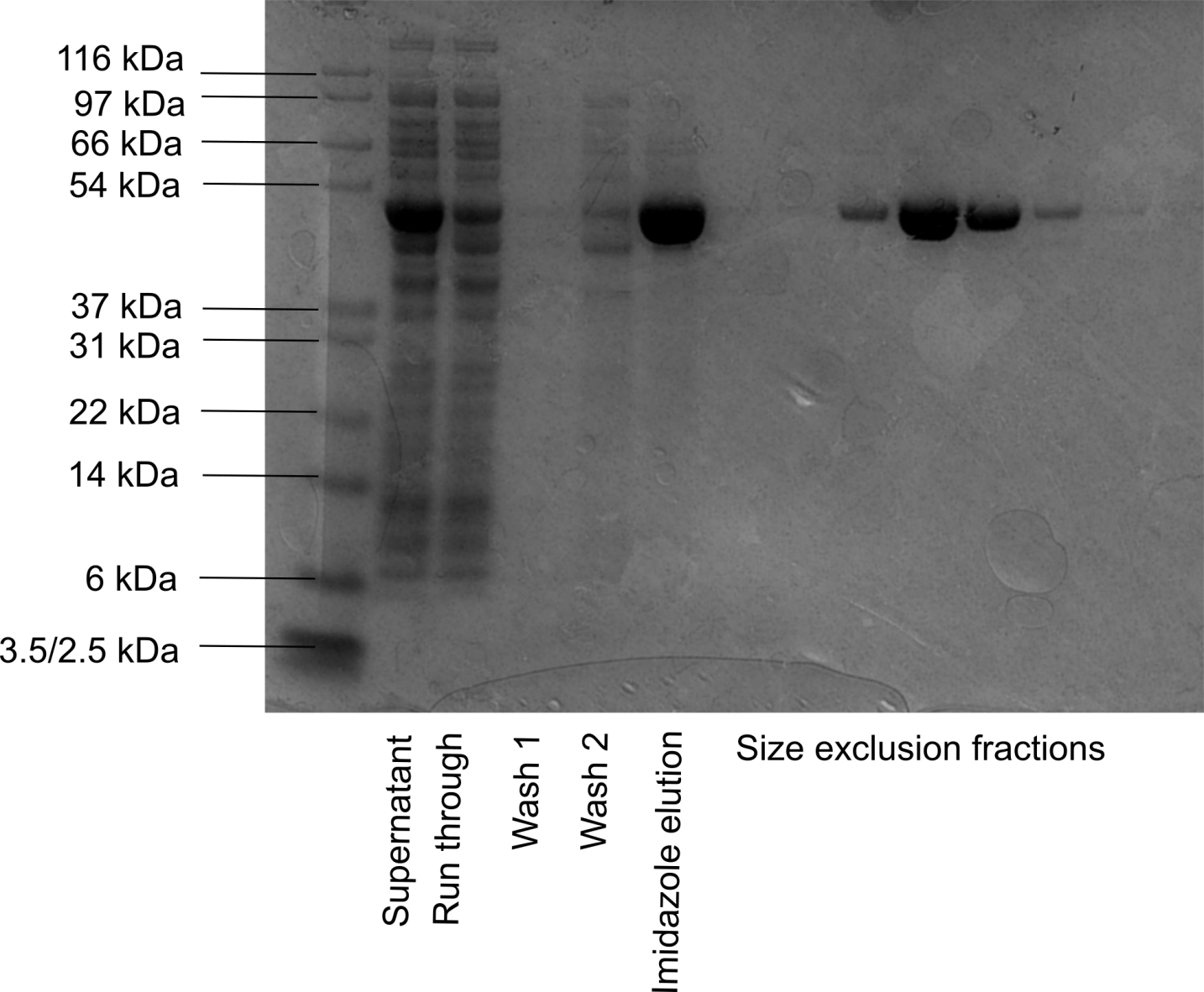


Figure S1. SDS-PAGE of *Bcel*PL6 His-tag purification from the supernatant and subsequent SEC purification (Superdex 75). Yield 30 mg L^−1^ culture.


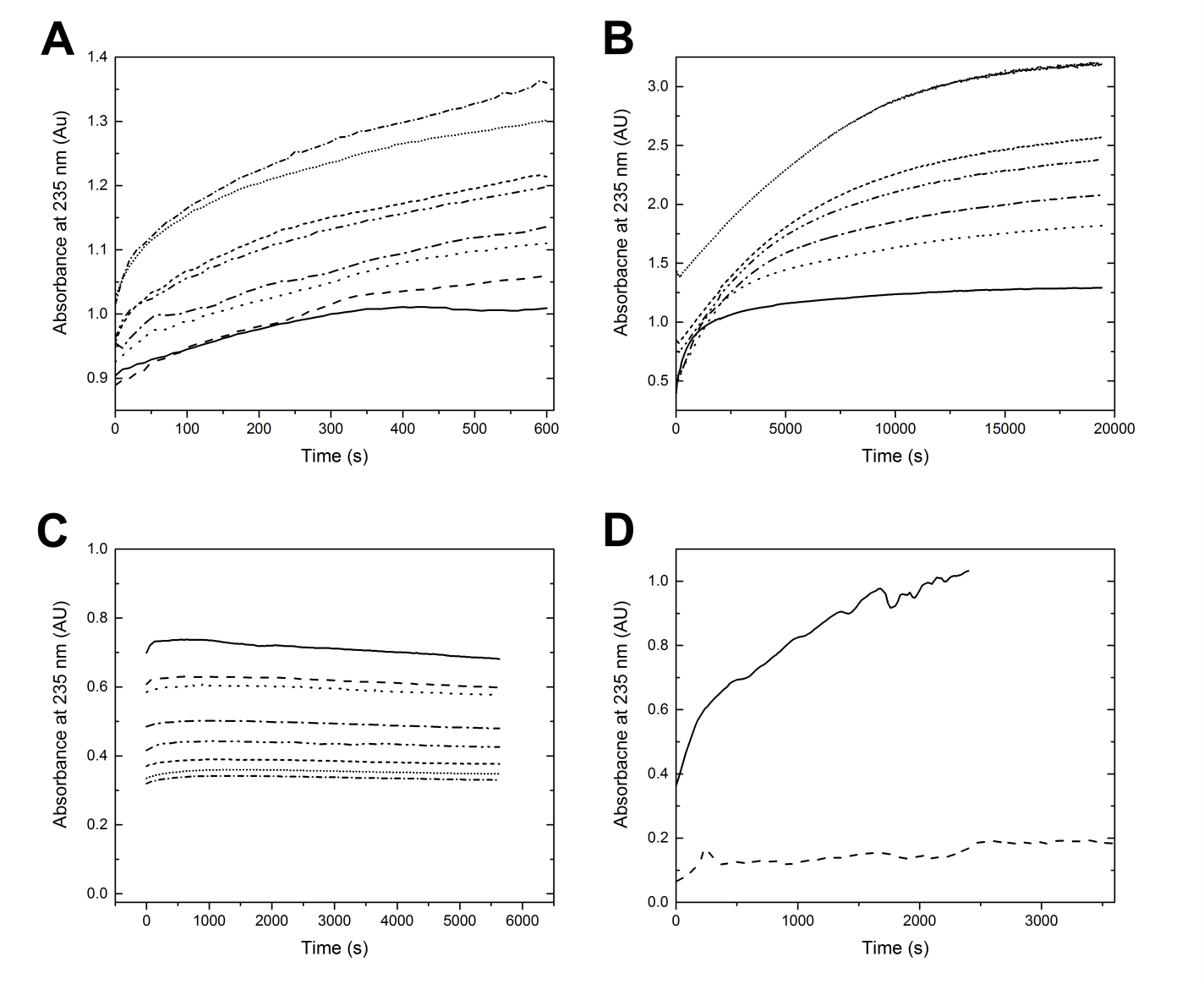


Figure S2. Progress of absorbance at 235 nm with increasing amounts of substrate (0.2, 0.4, 0.6, 0.8, 1.0, 1.2, 1.5 and 2 mg mL^−1^) incubated at 37 °C. A) 6 µM *Bcel*PL6 with polyG B) 100 nM *Bcel*PL6 with polyMG, C) 100 nM *Bcel*PL6 with acetylated polyM and D) 100 nM *Bcel*PL6 with 2 mM DP8M (solid line) and 2 mM NaBH_4_ reduced DP8M (dashed line).


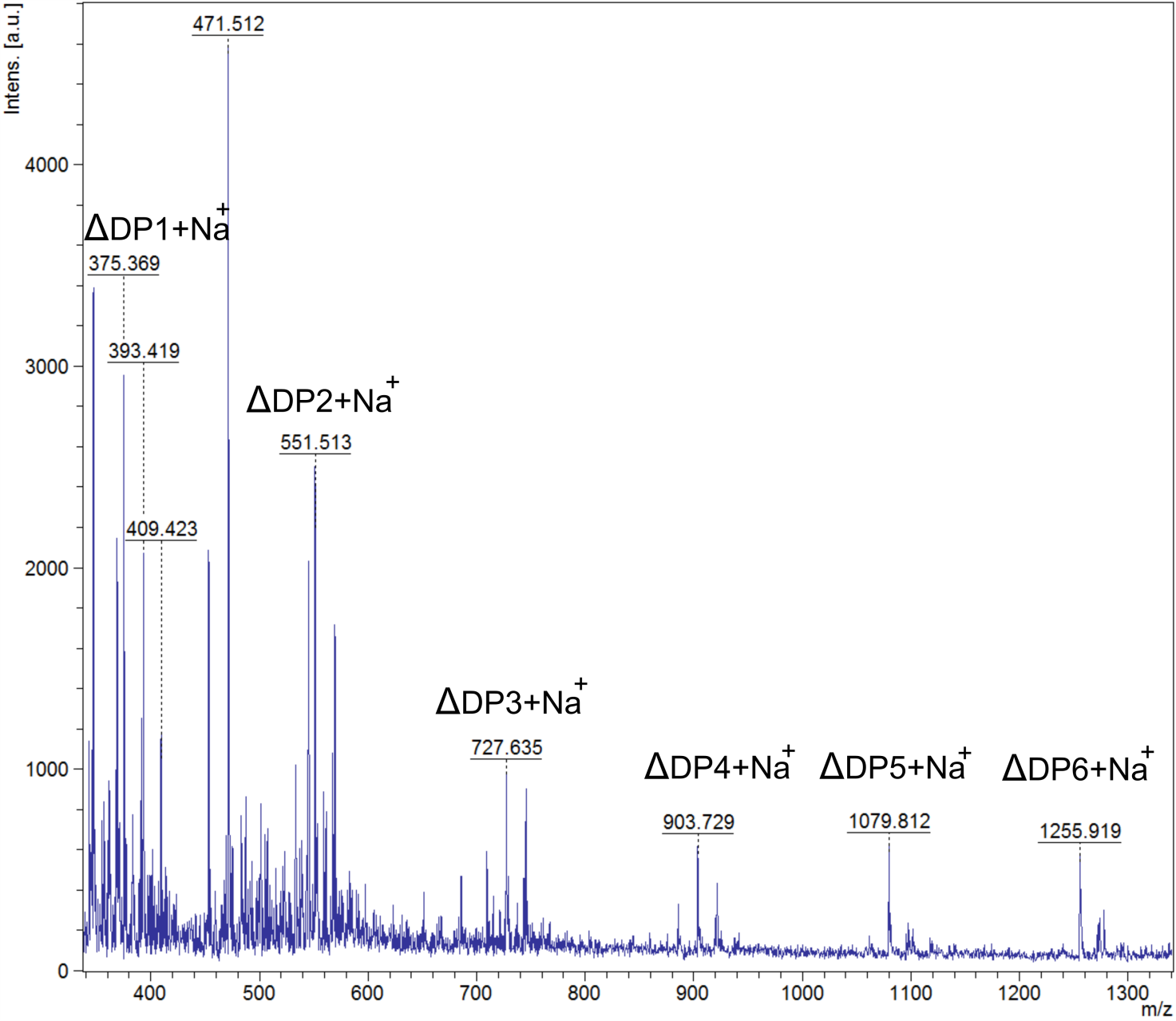


Figure S3. Linear positive MALDI-TOF of 4 mg mL^-1^ alginate incubated with 100 nM *Bcel*PL6 for 2 h at 37°C. The assigned peaks correspond to an unsaturated disaccharide sodium adduct (ΔDP1+Na^+^), an unsaturated trisaccharide sodium adduct (ΔDP2+ Na^+^), and so forth. Unassigned peaks are either noise or matrix peaks.


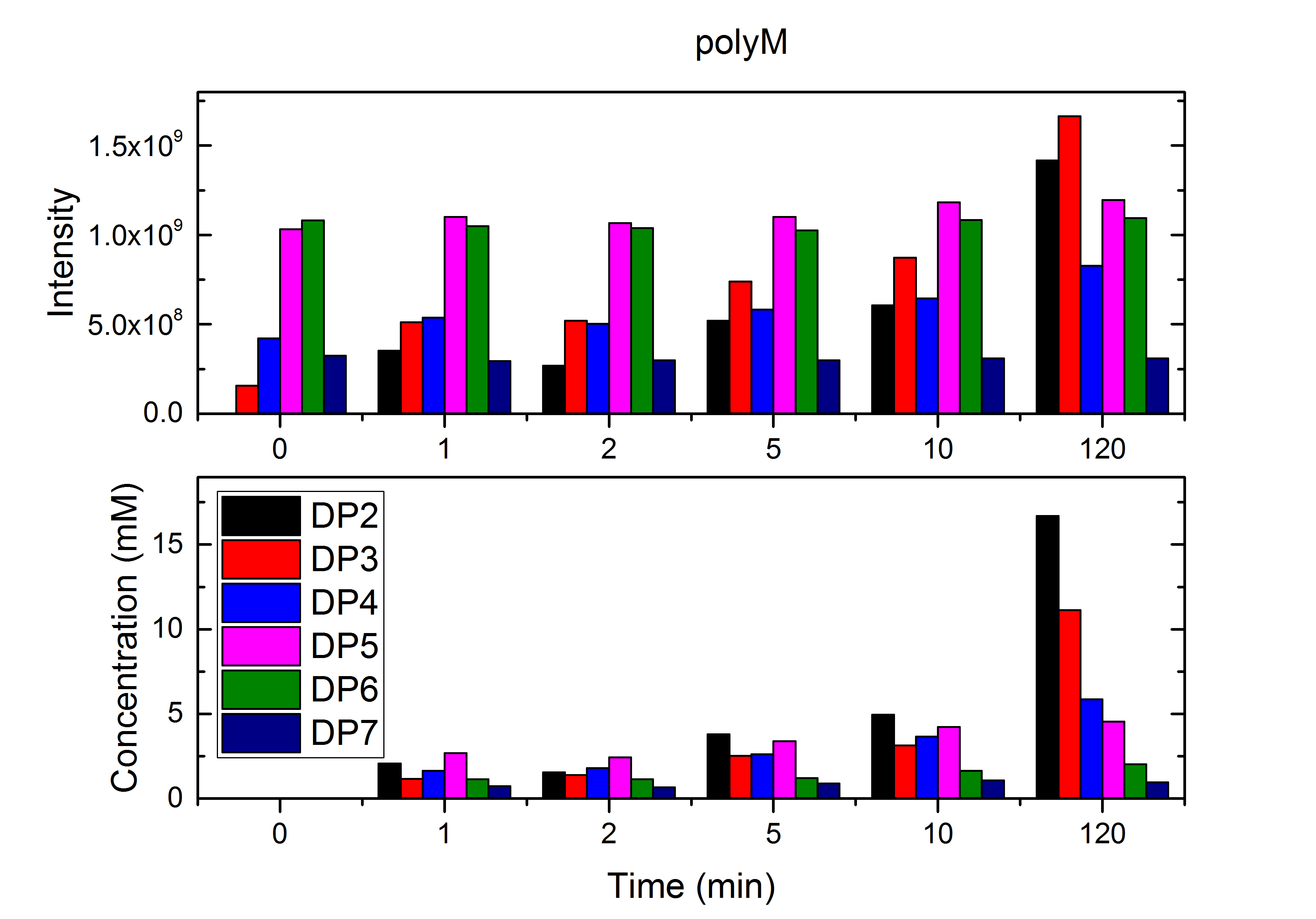


Figure S4. LC-ESI-MS analysis of breakdown products of polyM by *Bcel*PL6. Top: Intensity signal of masses as a function of time. Bottom: Integral of absorbance at 235 nm as a function of time converted to mM using ε = 6150 M^-1^ cm^-1^. Integral results can be found also in Table S1.

Table S1. Progress of product formation by *Bcel*PL6 acting on 20 mg mL^-1^ substrate based on HPLC and absorbance at 235 nm. Percentages are given as w/w. That the final numbers do not add up to 100 % is likely due to the definition of the border of the integration of the chromatogram.

| **Time (min)** | **DP2 (mM)** | **DP3 (mM)** | **DP4 (mM)** | **DP5 (mM)** | **DP6 (mM)** | **DP7 (mM)** |  |
| --- | --- | --- | --- | --- | --- | --- | --- |
| **Alginate** |  |  |  |  |  |  |  |
| 0 | 0.00 | 0.00 | 0.00 | 0.00 | 0.00 | 0.00 |  |
| 1 | 1.59 | 0.00 | 2.84 | 0.46 | 3.77 | 0.20 |  |
| 2 | 2.41 | 0.00 | 3.42 | 0.51 | 4.01 | 0.28 |  |
| 5 | 4.98 | 0.31 | 5.37 | 0.82 | 4.36 | 0.53 |  |
| 10 | 7.80 | 0.65 | 7.75 | 1.24 | 4.65 | 0.90 |  |
| 30 | 14.55 | 1.09 | 9.75 | 1.67 | 2.49 | 1.01 |  |
| 60 | 22.10 | 1.62 | 9.08 | 1.89 | 1.80 | 1.13 |  |
| 120 | 29.90 | 2.17 | 5.72 | 1.80 | 1.84 | 0.83 |  |
| **polyM** |  |  |  |  |  |  |  |
| 0 | 0.00 | 0.00 | 0.00 | 0.00 | 0.00 | 0.00 |  |
| 1 | 2.07 | 1.16 | 1.64 | 2.68 | 1.15 | 0.73 |  |
| 2 | 1.54 | 1.40 | 1.81 | 2.44 | 1.14 | 0.66 |  |
| 5 | 3.79 | 2.53 | 2.61 | 3.40 | 1.22 | 0.89 |  |
| 10 | 4.95 | 3.14 | 3.66 | 4.23 | 1.65 | 1.08 |  |
| 120 | 16.70 | 11.13 | 5.85 | 4.55 | 2.03 | 0.95 |  |
|  | **DP2 (%)** | **DP3 (%)** | **DP4 (%)** | **DP5 (%)** | **DP6 (%)** | **DP7 (%)** | **sum (%)** |
| **Alginate** |  |  |  |  |  |  |  |
| 0 | 0.01 | 0.01 | 0.01 | 0.01 | 0.02 | 0.02 | 0.07 |
| 1 | 2.79 | 0.01 | 9.99 | 2.04 | 19.90 | 1.23 | 35.96 |
| 2 | 4.25 | 0.01 | 12.04 | 2.26 | 21.19 | 1.71 | 41.46 |
| 5 | 8.76 | 0.82 | 18.89 | 3.62 | 23.00 | 3.25 | 58.35 |
| 10 | 13.73 | 1.72 | 27.30 | 5.44 | 24.57 | 5.56 | 78.33 |
| 30 | 25.61 | 2.86 | 34.31 | 7.35 | 13.13 | 6.21 | 89.47 |
| 60 | 38.90 | 4.28 | 31.96 | 8.32 | 9.49 | 6.99 | 99.95 |
| 120 | 52.62 | 5.72 | 20.13 | 7.93 | 9.71 | 5.11 | 101.23 |
| **polyM** |  |  |  |  |  |  |  |
| 0 | 0.01 | 0.00 | 0.01 | 0.00 | 0.00 | 0.02 | 0.03 |
| 1 | 3.64 | 3.07 | 5.76 | 11.81 | 6.08 | 4.51 | 34.86 |
| 2 | 2.71 | 3.70 | 6.35 | 10.74 | 6.01 | 4.04 | 33.55 |
| 5 | 6.66 | 6.67 | 9.18 | 14.96 | 6.42 | 5.51 | 49.40 |
| 10 | 8.72 | 8.28 | 12.87 | 18.63 | 8.70 | 6.67 | 63.87 |
| 120 | 29.39 | 29.39 | 20.60 | 20.03 | 10.73 | 5.87 | 116.01 |


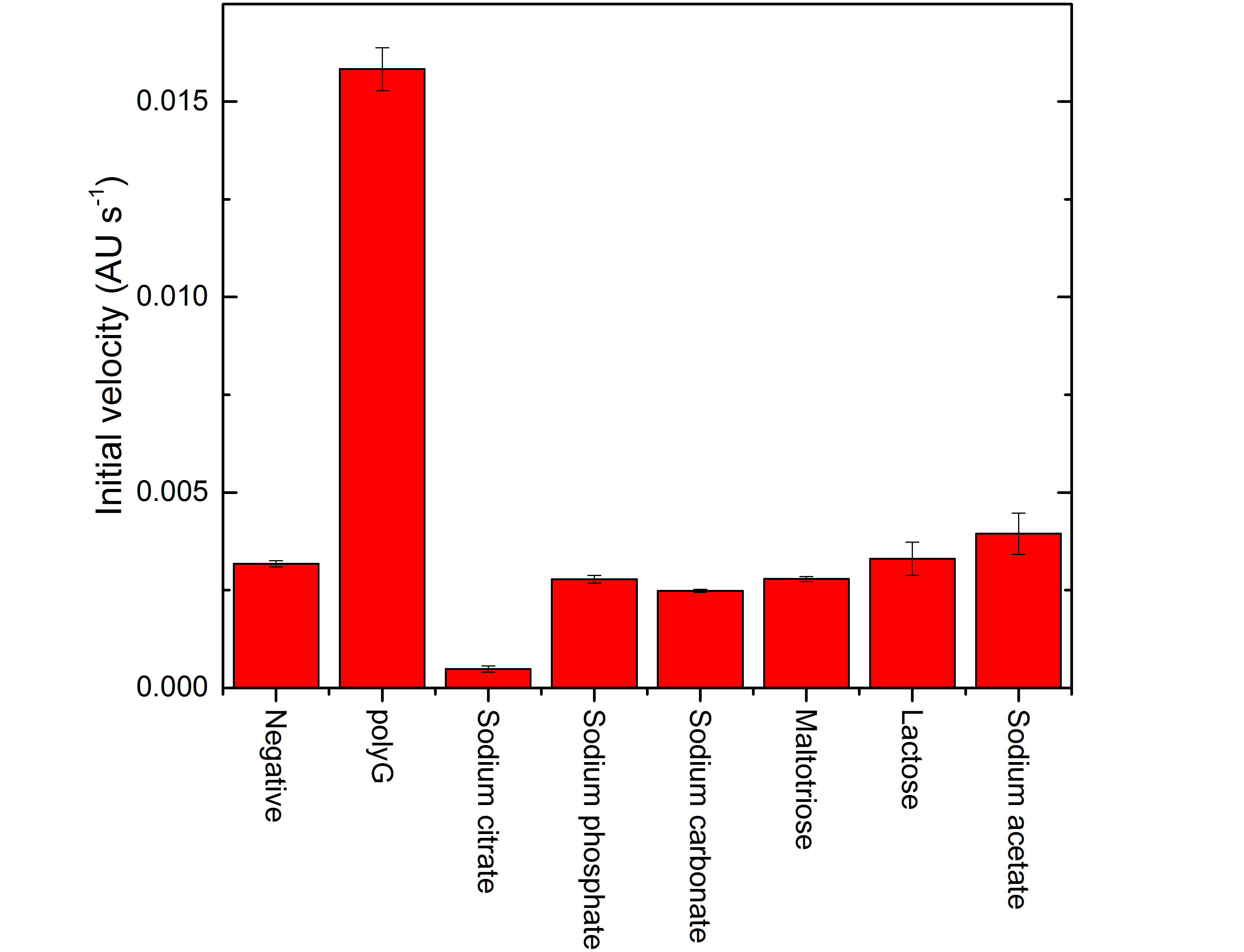


Figure S5. Effect of adding 2 mg mL^-1^ of polyG, 0.78 mM sodium citrate, 12.2 mM sodium phosphate, 18.9 mM sodium carbonate, 4.0 mM maltrotriose, 5.8 mM lactose or 24.3 mM sodium acetate (or 2 mg mL^-1^of all compounds) to a reaction of 1 mg mL^-1^ alginate and 100 nM *Bcel*Pl6.

^
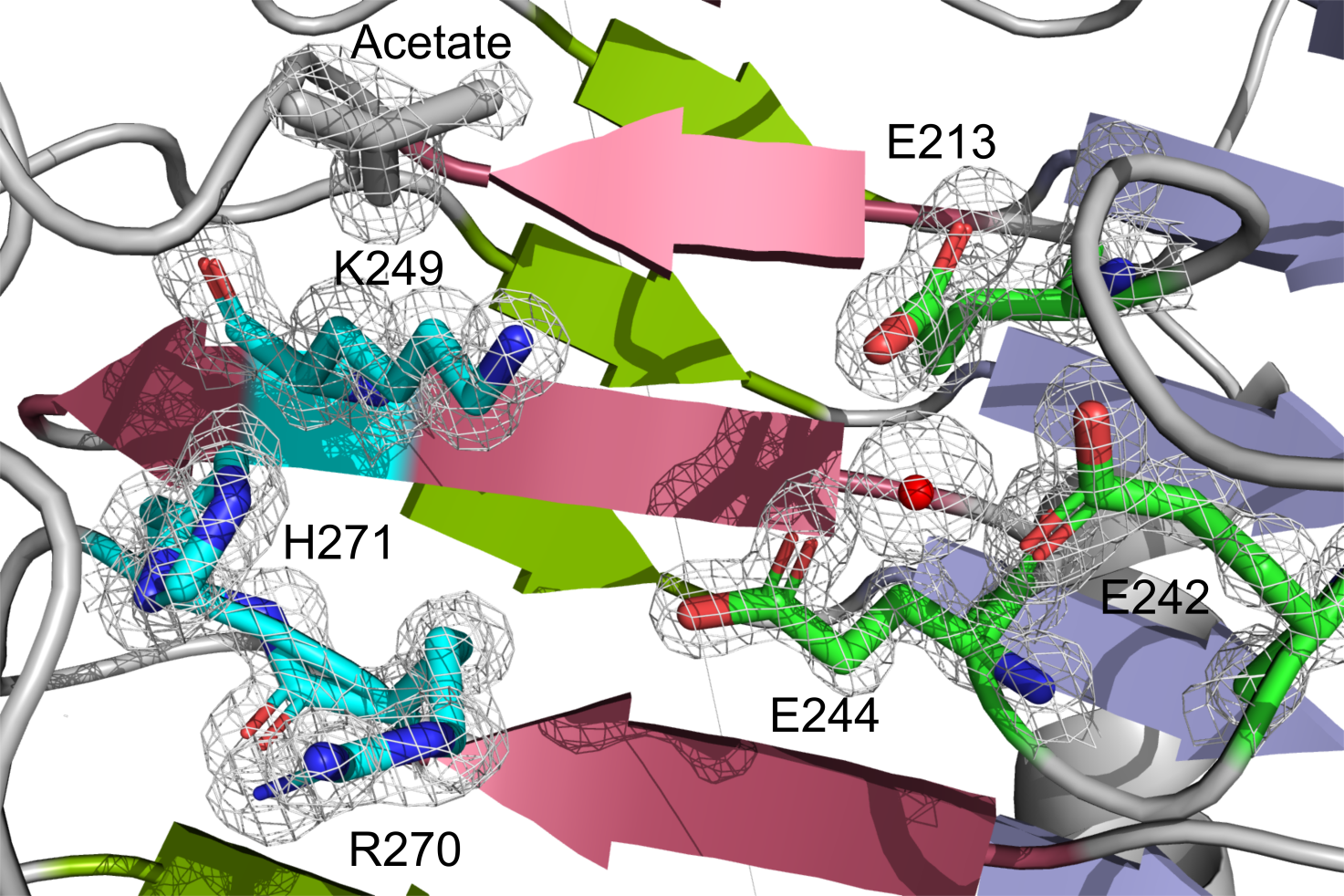
^

Figure S6. Electron density (sigma 2.0) of catalytic (cyan) and calcium coordinating (green) residues. Calcium (red) and the acetate (grey) present in the active site of *Bcel*PL6.

Table S2. Conservation analysis of primary structures of PL1, PL6, PL9 and alginate epimerases. Residues which are part of an asparagine ladder (red), catalytic residues (green) as well as residues not part of the features (black) are listed. Numbers in brackets indicate the percentage of conservation. See Experimental for alignment programs and software.

| **Protein** | **Family** | **Sequences retrieved** | **Sequences**  **aligned** | **Sequence**  **identify** | **±** | **No. Asn**  **≥99%**  **conserved** | **Residues**  **≥99%**  **conservation** | **Residues**  **≥98%**  **conservation** |
| --- | --- | --- | --- | --- | --- | --- | --- | --- |
| ***Bcel*PL6** | PL6 | 2089 | 1944 | 22.6 | 4.6 | 6 | G46  N234  E244  K249  N258 (100%) R270  G272  N280 (100%) G292  N305  N350  N376 | N199  F201  G218  H271(95%)  R294  G449  N402 (93%) |
| **1PCL** | PL1 | 11882 | 2581 | 30.6 | 8.6 | 1 | G21  G86  D153  D177  T186  K197  G202  R230  P232  R235  N244 | ND |
| **1RU4** | PL9 | 4842 | 3587 | 26.0 | 4.9 | 1 | G178  D209  G210  N231  D233  D234  G235  D237  G271  G275  G297 | ND |
| **4NK6** | Alginate  epimerase | 1882 | 1379 | 63.1 | ± | 5 | R228  G250 (100%)  G258  G292  Y314  D317  P318  H319  N330  H339  G340  I342  N355  N360  G364  N379 (100%)  G388 (100%)  S394  N402  N407  G411  N425 | ND |

Table S3. Conservation of key residues affecting activity in AlyGC

(ref: 27 PDB: 5GKD) in the structure of *Bcel*PL6

| *Bcel*PL6 | *Bcel*PL6 position | AlyGC | Effect of mutation in AlyGC (27) |
| --- | --- | --- | --- |
| R | 160 | Y | Y130A loses activity |
| N | 210 | N | N181A loses activity |
| E | 214 | E | E184A has almost no activity |
| R | 216 | R | R187A loses activity |
| E | 242 | E | E213A is inactive |
| E | 244 | E | E215A is inactive |
| K | 249 | K | K220A is inactive |
| R | 270 | R | R241A is inactive |
| H | 271 | H | H242A no effect |
| R | 294 | R | R265A has almost no activity |
| Y | 339 | Y | Y304A has almost no activity |


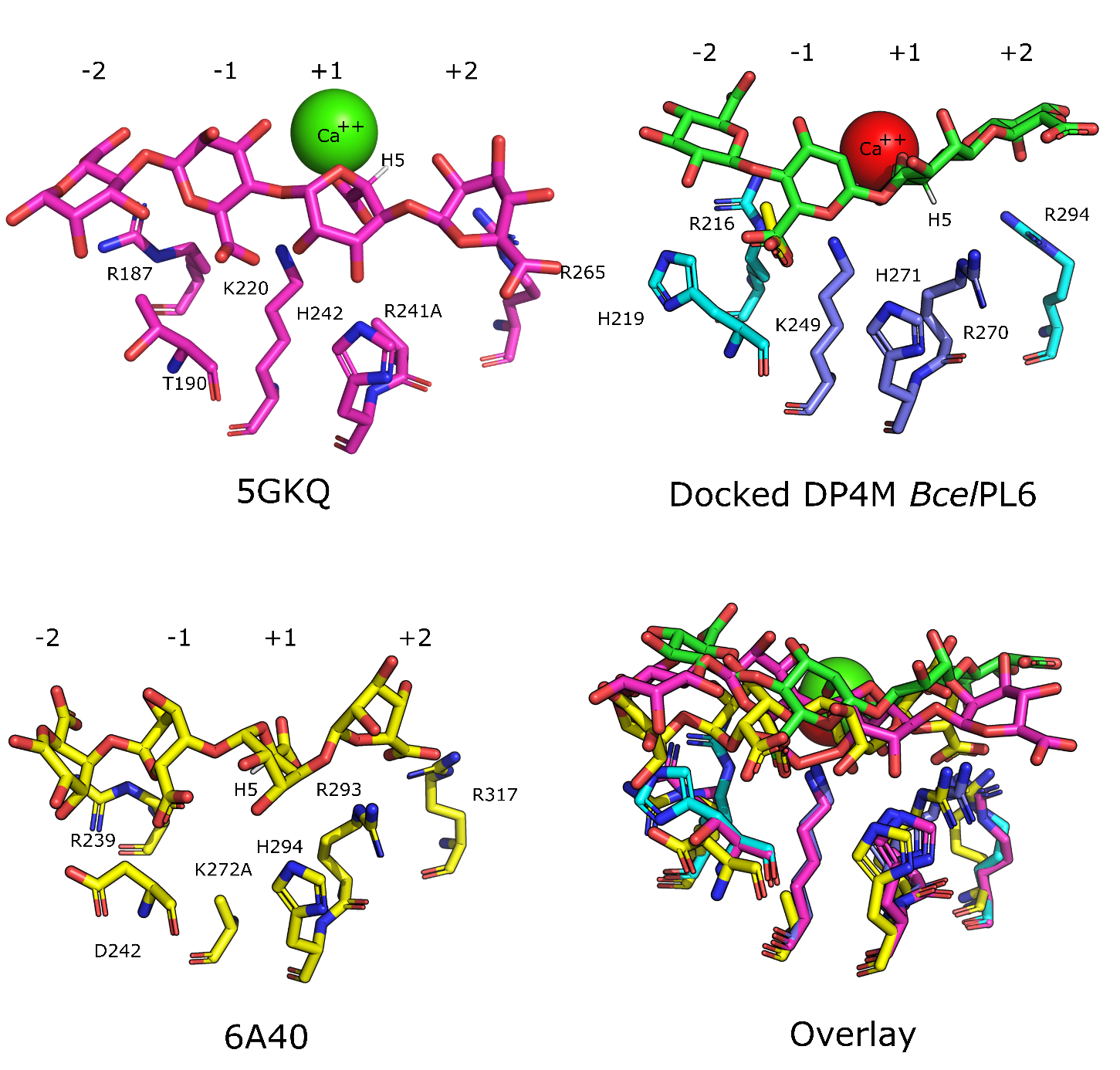


Figure S7: Details of the active site of AlyGC (5GKQ) with bound DP4M, of AlyF (6A40) with bound DP4G substrate, and of *Bcel*PL6 (6QPS) with docked DP4M The yellow molecule is an acetate found in the active site of *Bcel*PL6 (6QPS). The overlay of the three structures are presented as well (blue: *Bcel*PL6, yellow: AlyF and purple: AlyGC).

Table S4. Mutagenesis primer sequence. Numbers in brackets represent the length of the primer.

| Primer name | Mutation | Sequence |
| --- | --- | --- |
| a745c_a747t_ anti | K249H | CCTGCGCAATACATTATGACAGGAATGCACAGAAATAATCTCATTTTCTCC (51) |
| a745c_a747t_ | K249H | GGAGAAAATGAGATTATTTCTGTGCATTCCTGTCATAATGTATTGCGCAGG (51) |
| c808t_g809a_ anti | R270Y | ATAATGCCCATGATAACAAACCAATCCACCGGCGGATTCA  (40) |
| c808t_g809a_ | R270Y | TGAATCCGCCGGTGGATTGGTTTGTTATCATGGGCATTAT  (40) |
| c811a_anti | R270A | ATCACATTATAATGCCCATTACGACAAACCAATCCACCG  (39) |
| c811a_ | R270A | CGGTGGATTGGTTTGTCGTAATGGGCATTATAATGTGAT  (39) |
| a685g_a686c | K249A | GGAGAAAATGAGATTATTTCTGTGGCATCCTGTCATAATGTATTGCGCAG (50) |
| a685g_a686c_anti | K249A | CTGCGCAATACATTATGACAGGATGCCACAGAAATAATCTCATTTTCTCC (50) |
| g457c_t459g_ anti | D153Q | GTAATCCGACCCAATATTCCTGTCCTTCATTCGGTCGTTCA  (41) |
| g457c_t459g_ | D153Q | TGAACGACCGAATGAAGGACAGGAATATTGGGTCGGATTAC  (41) |
| t463a_a464g_ anti | Y155S | GTAATCCGACCCAACTTTCATCTCCTTCATTCGGTCGTTCA  (41) |
| t463a_a464g_ | Y155S | TGAACGACCGAATGAAGGAGATGAAAGTTGGGTCGGATTAC  (41) |
| c478t_g479a_ g480t_ | R160Y | AGGAGATGAATATTGGGTCGGATTATATGGAACTAATAACCGGATAGACC (50) |
| c478t_g479a_ g480t_anti | R160Y | GGTCTATCCGGTTATTAGTTCCATATAATCCGACCCAATATTCATCTCCT  (50) |
| g529a_g530a _c531g_anti | G177K | ACACCTGCAACACCAGTCCCTTTACTCTTTTATTAGCAAAGTAACAATGG (50) |
| g529a_g530a _c531g_ | G177K | CCATTGTTACTTTGCTAATAAAAGAGTAAAGGGACTGGTGTTGCAGGTGT (50) |
| _c862a_anti | H271N | ATCACATTATAATGCCATTACGACAAACCAATCCACCG  (39) |
| _c862a_ | H271N | CGGTGGATTGGTTTGTCGTAATGGGCATTATAATGTGAT  (39) |
| t550a_anti | W184R | TATCAGCACTCAGCCTCACCTGCAACACCAG  (31) |
| t550a_ | W184R | CTGGTGTTGCAGGTGAGGCTGAGTGCTGATA  (31) |
| t1012a_c1013 g_anti | S338R | TGAAATTGGAGCCGTTAACTAGATACCATCGGGTAGAGAATG  (42) |
| t1012a_c1013 g_ | S338R | CATTCTCTACCCGATGGTATCTAGTTAACGGCTCCAATTTCA  (42) |
| c655a_a 656c_ | H219T | CAGAAATTATTCGTATCGGGACTTCCTGGTCTTCCCAATTGG  (42) |
| c655a_a 656c_anti | H219T | CCAATTGGGAAGACCAGGAAGTCCCGATACGAATAATTTCTG  (42) |
